# Type I interferon signaling induces a delayed antiproliferative response in respiratory epithelial cells during SARS-CoV-2 infection

**DOI:** 10.1128/jvi.01276-23

**Published:** 2023-11-17

**Authors:** Juliana Bragazzi Cunha, Kyle Leix, Emily J. Sherman, Carmen Mirabelli, Tristan Frum, Charles J. Zhang, Andrew A. Kennedy, Adam S. Lauring, Andrew W. Tai, Jonathan Z. Sexton, Jason R. Spence, Christiane E. Wobus, Brian T. Emmer

**Affiliations:** 1 Department of Internal Medicine, University of Michigan Medical School, Ann Arbor, Michigan, USA; 2 Department of Microbiology and Immunology, University of Michigan Medical School, Ann Arbor, Michigan, USA; 3 Department of Medicinal Chemistry, College of Pharmacy, University of Michigan, Ann Arbor, Michigan, USA; 4 VA Ann Arbor Healthcare System, Ann Arbor, Michigan, USA; 5 Department of Cell and Developmental Biology, University of Michigan Medical School, Ann Arbor, Michigan, USA; 6 Department of Biomedical Engineering, University of Michigan College of Engineering, Ann Arbor, Michigan, USA; University of North Carolina, Chapel Hill, North Carolina, USA

**Keywords:** CRISPR screen, SARS-CoV-2, interferon, cell proliferation

## Abstract

Disease progression during SARS-CoV-2 infection is tightly linked to the fate of lung epithelial cells, with severe cases of COVID-19 characterized by direct injury of the alveolar epithelium and an impairment in its regeneration from progenitor cells. The molecular pathways that govern respiratory epithelial cell death and proliferation during SARS-CoV-2 infection, however, remain unclear. We now report a high-throughput CRISPR screen for host genetic modifiers of the survival and proliferation of SARS-CoV-2-infected Calu-3 respiratory epithelial cells. The top four genes identified in our screen encode components of the same type I interferon (IFN-I) signaling complex*—IFNAR1*, *IFNAR2*, *JAK1*, and *TYK2*. The fifth gene, *ACE2*, was an expected control encoding the SARS-CoV-2 viral receptor. Surprisingly, despite the antiviral properties of IFN-I signaling, its disruption in our screen was associated with an increase in Calu-3 cell fitness. We validated this effect and found that IFN-I signaling did not sensitize SARS-CoV-2-infected cultures to cell death but rather inhibited the proliferation of surviving cells after the early peak of viral replication and cytopathic effect. We also found that IFN-I signaling alone, in the absence of viral infection, was sufficient to induce this delayed antiproliferative response in both Calu-3 cells and iPSC-derived type 2 alveolar epithelial cells. Together, these findings highlight a cell autonomous antiproliferative response by respiratory epithelial cells to persistent IFN-I signaling during SARS-CoV-2 infection. This response may contribute to the deficient alveolar regeneration that has been associated with COVID-19 lung injury and represents a promising area for host-targeted therapeutic development.

**IMPORTANCE:**

The proliferation of respiratory epithelial cells is crucial to host recovery from acute lung injury caused by SARS-CoV-2 and other viral pathogens, but the molecular pathways that govern this process are poorly understood. We performed a high-throughput CRISPR screen that surprisingly revealed a detrimental effect of specific host response, type I interferon (IFN-I) signaling, on the fitness of SARS-CoV-2-infected Calu-3 cells. While IFN-I signaling has been previously associated with several potential downstream responses, we found this effect to be primarily mediated by an inhibition of Calu-3 cellular proliferation after the early peak of SARS-CoV-2-induced cell death. Our findings provide a plausible mechanism for how sustained IFN-I signaling during SARS-CoV-2 infection might worsen lung pathology by blocking the regeneration of the alveolar epithelium from progenitor cells.

## INTRODUCTION

The respiratory epithelium plays an important role during COVID-19, as alveolar epithelial cells express the SARS-CoV-2 receptor ACE2 and support active viral replication during infection (1, 2). Diffuse alveolar damage, characterized by the injury of epithelial cells, compromised barrier function, and leakage of proteinaceous exudate into the alveolar space, is a histopathologic hallmark of severe COVID-19 and other causes of acute respiratory distress syndrome (ARDS) (1, 3). Injury to the alveolar epithelium is counterbalanced by a regenerative pathway involving the proliferation and trans-differentiation of type 2 alveolar epithelial (AT2) cells that repopulate the alveolar lining and resorb fluid from the alveolar space (4–7). COVID-19 has been associated with a deficiency in this AT2 regenerative response (8, 9), though the molecular basis for this deficiency is unknown.

Severe cases of COVID-19 typically develop seven or more days after initial infection (10), at which time an exuberant host response, rather than uncontrolled viral replication, appears to drive lung pathology. Accordingly, virus-targeted therapies such as remdesivir have little or no benefit in severe COVID-19 (11, 12), while host-targeted therapies such as corticosteroids and Janus kinase inhibitors have been shown to improve clinical outcomes (13–16). These treatments modulate several host signaling pathways, however, and it is unknown which are responsible for their therapeutic effect.

The type I interferon (IFN-I) response plays a pivotal role in numerous infections, causing pleiotropic effects with the potential to be either protective or detrimental to the host. IFN-I signaling may induce a cellular antiviral state, modulate host innate and adaptive immunity, and regulate pathways of programmed cell death and cellular proliferation. The relative contributions of these effects and their net impact on infection are highly dependent on the specific pathogen and cell type involved (17). For SARS-CoV-2 infection, studies in animal models have suggested that the net impact of systemic IFN-I signaling is detrimental to the host (18–20). However, since these studies involve systemic modulation of IFN-I signaling, it is unclear which cell type(s) and downstream response(s) are responsible for this pathogenic effect. The precise role of IFN-I signaling during SARS-CoV-2 infection remains enigmatic and likely depends on the timing and magnitude of the response (21).

Over the past decade, CRISPR screening has emerged as a powerful tool for detecting functional interactions between host cells and viral pathogens (22). Since the onset of the COVID-19 global pandemic, several groups have applied this approach to SARS-CoV-2 infection (23–33). Although some genes have been consistently identified across multiple studies, many have shown limited overlap, potentially due to differences in cell type or other variables in screen design. For most identified host factors, their mechanism of effect during viral infection is unknown.

We now report the results of a CRISPR screen for host factors affecting SARS-CoV-2 infection in Calu-3 cells, a cell line of respiratory epithelial origin that recapitulates the SARS-CoV-2 replication kinetics and interferon responses of primary human airway cells (34). The findings from our screen and subsequent mechanistic studies implicate IFN-I signaling as having a pathogenic effect on respiratory epithelial cells during SARS-CoV-2 infection due to their induction of a delayed antiproliferative response that limits their recovery after the initial peak of viral replication and cell death.

## RESULTS

### CRISPR screen for modifiers of Calu-3 cell fitness during SARS-CoV-2 infection

**Fig 1:**
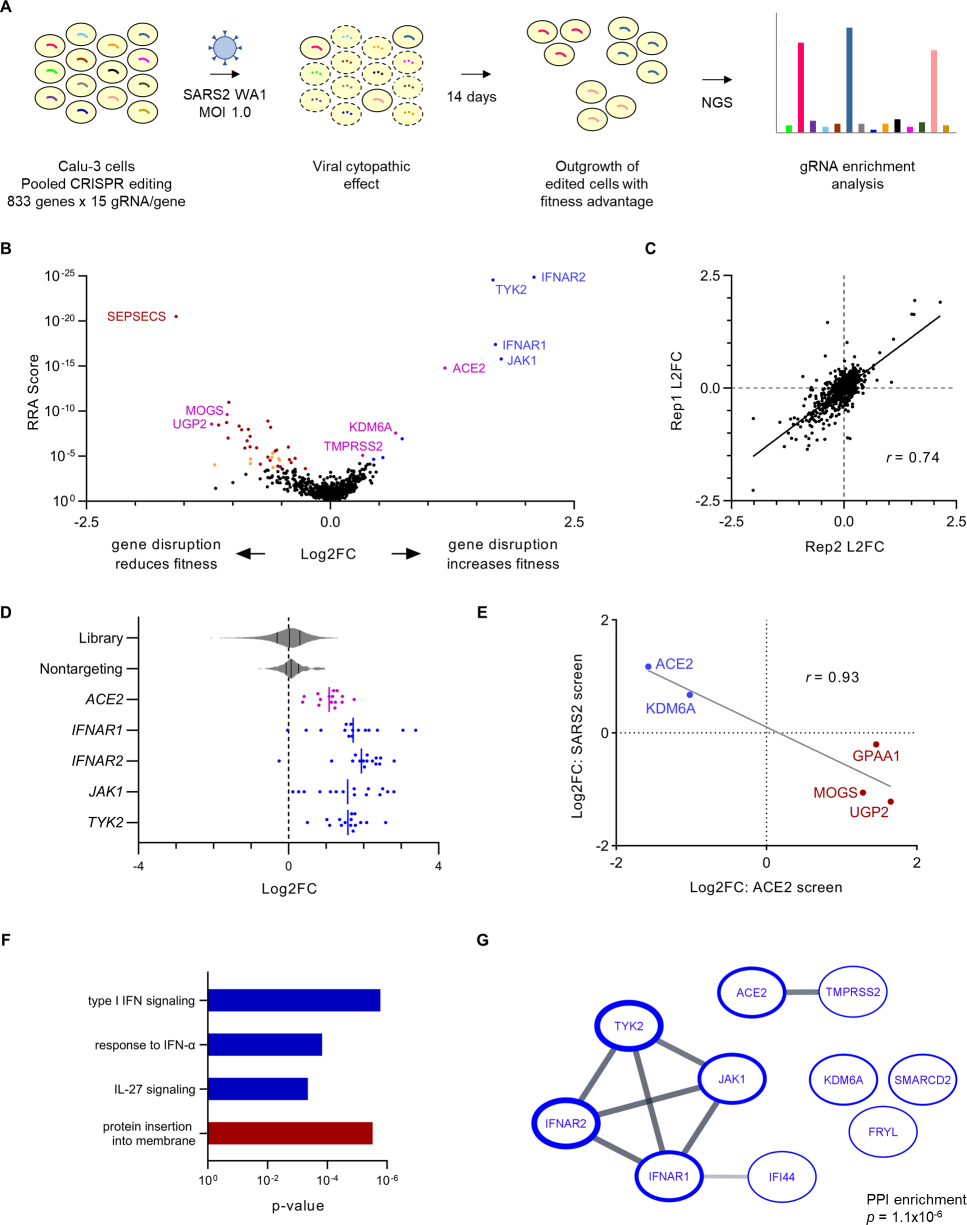
CRISPR screen for modifiers of Calu-3 cell fitness during SARS-CoV-2 infection. (**A**) Schematic of CRISPR screening strategy. (**B**) Volcano plot of CRISPR screen results. Genes whose disruption confers a significant [false discovery rate (FDR) <5%] increase or decrease in cell fitness during SARS-CoV-2 infection are highlighted in blue or red, respectively. Filtered genes whose disruption confers a significant effect on cell fitness in the absence of viral infection (Fig. S3) are shaded in orange. Identified genes with a known role in SARS-CoV-2 entry or ACE2 regulation are highlighted in magenta. Select genes are labeled, and full-screen results are provided in Tables S1 and S2. (**C**) Pairwise correlation between independent biologic replicates for the aggregate log2 fold change of all gRNAs targeting a given gene. The correlation coefficient was calculated by the Pearson method. (**D**) Individual gRNA log2 fold change for every 15 gRNAs targeting the indicated gene, with violin plots showing the distribution of changes for the entire library and the subset of control nontargeting gRNAs. The vertical lines indicate the mean of individual gRNAs for each gene. (**E**) Correlation between aggregate gRNA log2 fold change in independent screens for modifiers of Calu-3 ACE2 surface abundance and cell fitness during SARS-CoV-2 infection. The genes whose disruption was associated with a decrease in surface ACE2 and an increase in cell fitness during SARS-CoV-2 infection are highlighted in blue. The genes whose disruption was associated with an increase in surface ACE2 and a decrease in cell fitness during SARS-CoV-2 infection are highlighted in red. The correlation coefficient was calculated by the Pearson method. (**F**) Functional annotations significantly enriched (*P* < 10^−3^) among identified genes whose disruption was associated with a significant increase (blue) or decrease (red) in Calu-3 cell fitness during SARS-CoV-2 infection. For multiple nodes within the same hierarchy, the annotation with the most significant enrichment was selected. (**G**) Established protein–protein interactions in the STRING database among genes identified in the screen whose disruption was associated with a significant increase in Calu-3 cell fitness during SARS-CoV-2 infection. The borders of individual nodes are weighed by the −log(RRA score) in the screen, and the lines connecting nodes are weighed by the strength of the protein–protein interaction within the STRING database. The significance of the number of protein–protein interactions relative to a randomly selected gene set was calculated by STRING.

We previously synthesized a high-resolution CRISPR library targeting 833 genes implicated in SARS-CoV-2 biology (35) based on their identification in prior CRISPR screens of SARS-CoV-2 cytopathic effect (23, 24, 26–30, 36), human genome-wide association studies of COVID-19 susceptibility (37), RNA-seq analysis of genes whose expression correlated with the SARS-CoV-2 receptor ACE2 (38), and our own genome-wide CRISPR screen for modifiers of ACE2 abundance (35). Our earlier application of this library to a flow cytometry–based screen led to our successful identification of cell type–specific modifiers of ACE2 surface abundance in HuH7 and Calu-3 cells (35). In this study, we repurposed this same library to screen for modifiers of Calu-3 cell fitness during SARS-CoV-2 infection (Fig. 1A). We generated pools of CRISPR-edited cells that we then infected with the WA1 strain of SARS-CoV-2. Immunofluorescence microscopy revealed that approximately 3% of Calu-3 cells were infected at 48 h, notably lower than predicted based on multiplicity of infection (MOI) calculations derived from the more permissive Vero E6 cell line (Fig. S1). SARS-CoV-2-infected Calu-3 cells began exhibiting signs of cytopathic effect at day 2 that became most prominent at approximately day 4 post-infection. Surviving cells were maintained in culture for 14 days post-infection to allow for the outgrowth of edited cells with a fitness advantage. We then quantified the abundance of each individual gRNA by massively parallel sequencing from genomic DNA isolated at days 0 and 14 post-infection and analyzed gRNA enrichment using MAGeCK (39) (Fig. 1B; Tables S1 and S2). Quality control analysis confirmed adequate depth of sampling (Fig. S2A) with high reproducibility between independent biologic replicates (Fig. 1C) and robust discrimination of the positive control *ACE2*-targeting and negative control nontargeting (NT) gRNAs in SARS-CoV-2-infected cells (Fig. 1D). To avoid identifying gene perturbations that had a general influence on host cell fitness independently of SARS-CoV-2 infection, we filtered out genes targeted by gRNAs that were significantly enriched or depleted in day 0 samples relative to the CRISPR library plasmid pool (Fig. S3A; Tables S3 and S4). The genes removed by this filtering strategy showed considerable overlap with those identified as serving a core essential function in an analysis of 1,070 cell lines (40) (Fig. S3B).

Overall, we identified 10 genes whose CRISPR targeting was associated with a significant (FDR < 5%) increase in Calu-3 cell fitness and 31 genes whose targeting was associated with a significant decrease in fitness during SARS-CoV-2 infection (Fig. 1B; Tables S1 and S2). As expected, the top hits of the former group included both *ACE2* and *TMPRSS2*, two genes with well-characterized roles in SARS-CoV-2 entry (41). Similarly, among the genes we previously identified as modifiers of ACE2 surface abundance in Calu-3 cells (35), we detected a strong inverse correlation between their effect on ACE2 and their effect on cell fitness during SARS-CoV-2 infection (Fig. 1E). This correlation supports both the validity of the screen and the relevance of quantitative changes in ACE2 abundance to cellular sensitivity to SARS-CoV-2 cytopathic effect.

In addition to *ACE2* and the ACE2 modifiers discussed above, we identified several other genes that influenced the fitness of SARS-CoV-2-infected Calu-3 cells. Ontology analysis revealed significant enrichment for a limited number of functional annotations, with the greatest effect observed for the type I interferon signaling pathway (Fig. 1F). An analysis of the STRING database likewise revealed significant enrichment in established protein–protein interactions (Fig. 1G; Fig. S4), most prominently among components of the type I interferon receptor signaling complex encoded by *IFNAR1*, *IFNAR2*, *JAK1*, and *TYK2*. Unexpectedly and in contrast to the canonical antiviral properties of the type I interferon response, the disruption of type I interferon signaling was associated with an increase in Calu-3 fitness, with gRNAs targeting these genes becoming enriched during SARS-CoV-2 infection (Fig. 1D). The aggregate magnitude of enrichment at 14 days post-infection for gRNAs targeting each of these genes was even greater than that observed for *ACE2* (Fig. 1D). For each gene, a significant enrichment was observed for multiple independent gRNAs, consistent with this effect being driven by on-target rather than off-target activity (Fig. 1D). These findings indicate that despite any potential antiviral effects of endogenous IFN-I signaling, its overall impact on SARS-CoV-2-infected populations in our screen led to a decrease in Calu-3 cell fitness.

### The overall effect of IFN-I signaling on SARS-CoV-2-infected Calu-3 cells is context-dependent

**Fig 2:**
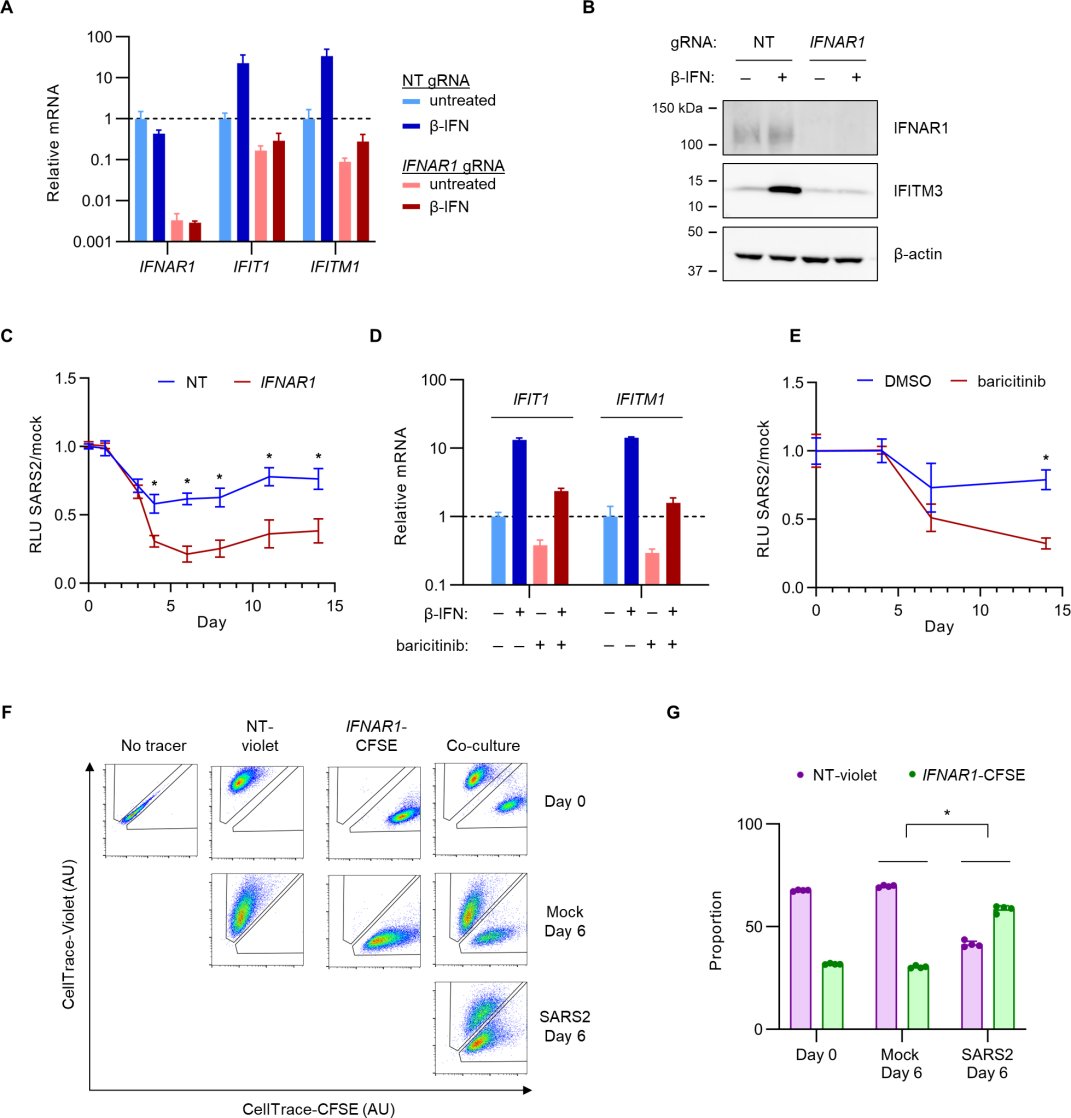
*IFNAR1*-disrupted Calu-3 cells exhibit reduced fitness during SARS-CoV-2 infection when cultured separately from control cells but increased fitness when cultured together. (**A–B**) Calu-3 cells were transduced with lentiviral constructs delivering Cas9 and either a *IFNAR1*-targeting or nontargeting control gRNA, followed by treatment with 1,000 U/mL exogenous β-IFN or vehicle control for 48 h and analysis of (**A**) mRNA by qRT-PCR with primer pairs for the indicated gene and (**B**) protein lysates by immunoblotting with antibodies for the indicated proteins. (**C**) Control and *IFNAR1*-disrupted cells were seeded in 96-well plates, infected with SARS-CoV-2 at MOI of 1 or mock-infected, and the resulting number of viable cells at indicated time points assayed by CellTiter-Glo luminescence. (**D**) Calu-3 cells were treated with 1,000 U/mL β-IFN or vehicle in the presence of 1 µM baricitinib or vehicle for 48 h and tested for induction of interferon-stimulated gene mRNA expression qRT-PCR. (**E**) Calu-3 cells were treated with 1 µM baricitinib or vehicle at the onset of infection with SARS-CoV-2 WA1 strain at MOI of 1. The number of viable cells at the indicated time points was monitored by CellTiter-Glo luminescence. (**F–G**) Calu-3 cells were treated with a nontargeting gRNA or *IFNAR1*-targeting gRNA, loaded with CellTrace-Violet or CellTrace-CFSE, respectively, and either maintained in isolation as gating controls or pooled together. The relative proportion of each cell type in the mixture at different time points after SARS-CoV-2 or mock infection was visualized (**F**) and quantified (**G**) by flow cytometry. All MOI estimates were derived from titering in Vero E6 cells. Error bars represent standard deviations of two to four replicates for each assay, and asterisks indicate *P* < 0.001 by Student’s *t*-test.

To validate our screen finding that IFN-I signaling decreased Calu-3 cell fitness during SARS-CoV-2 infection and to explore the mechanism for this effect, we next targeted the endogenous *IFNAR1* locus of Calu-3 cells by CRISPR and confirmed the efficient depletion of *IFNAR1* mRNA (Fig. 2A) (consistent with nonsense-mediated decay of transcripts with frameshift-causing indels) and IFNAR1 protein (Fig. 2B). We also confirmed that *IFNAR1*-disrupted cells were resistant to the induction of interferon-stimulated gene expression by exogenous β-IFN at a concentration approximating the endogenous response of SARS-CoV-2-infected wild-type Calu-3 cells (34) (Fig. 2A and B). Paradoxically, despite the protective effect of *IFNAR1* disruption in our screen, testing of these *IFNAR1*-disrupted cells in parallel to control cells revealed a greater decrease in viable cells during SARS-CoV-2 infection (Fig. 2C). A similar sensitization to SARS-CoV-2 cytopathic effect was observed for pharmacologic pre-treatment with a Janus kinase inhibitor, baricitinib, that inhibited IFN-I signaling in Calu-3 cells (Fig. 2D and E).

The discrepancy between our screen findings and our subsequent validation studies led us to consider the differences in experimental design between these approaches. In the screen, *IFNAR1*-disrupted cells shared the same extracellular environment as neighboring cells with intact IFN-I signaling, whereas in single gene disruption studies, each population occupied a separate physical space with its own conditioned media. To test whether this difference might underlie the discordance in our findings, we next tested control and *IFNAR1*-disrupted cells in a competitive co-culture experiment. We loaded each cell type with a different fluorescent dye and tracked their relative proportions over the course of SARS-CoV-2 or mock infection by flow cytometry. Consistent with our screen and in contrast to the observations with separate cultures, we found that *IFNAR1*-disrupted cells in this context exhibited a significant competitive advantage over control cells during SARS-CoV-2 infection but not during mock infection (Fig. 2F and G; Fig. S5). This competitive advantage for *IFNAR1*-disrupted cells was not mediated by the fluorescent dyes themselves, as it was observed with either combination of cell population and dye (Fig. S6). Together, these findings validate the detection of IFN-I signaling genes as modifiers of Calu-3 cell fitness in our pooled screen but indicate that the protective effect of IFN-I signaling disruption is dependent on the presence of neighboring cells with intact IFN-I signaling.

### The early IFN-I response protects SARS-CoV-2-infected Calu-3 cells by restricting viral replication

**Fig 3:**
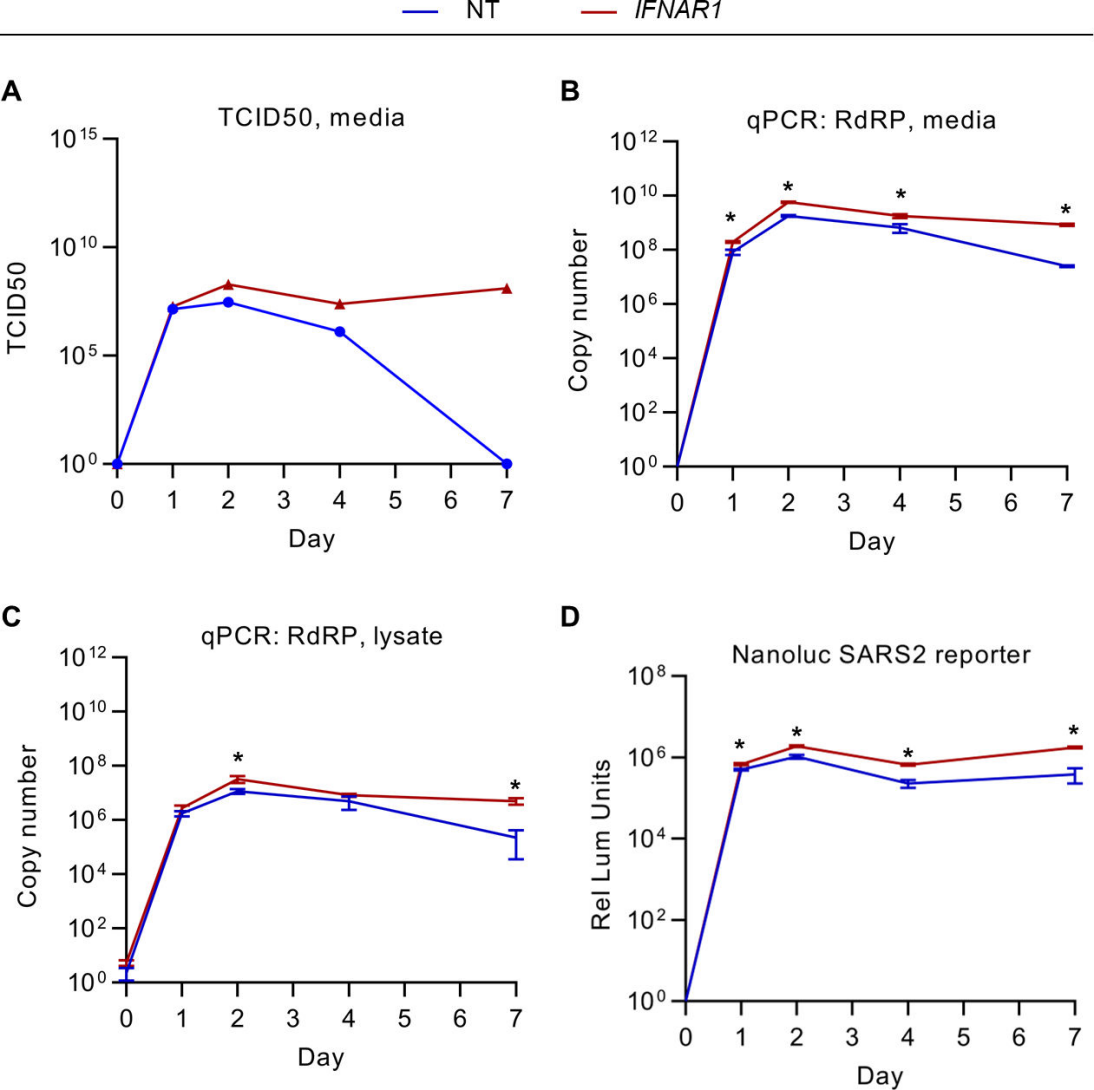
Early IFN-I signaling restricts SARS-CoV-2 replication in Calu-3 cells. (**A–C**) Control nontargeting and *IFNAR1*-disrupted cells were infected with SARS-CoV-2 WA strain at MOI of 1 (as derived from titering in Vero E6 cells) and cellular lysates and conditioned media collected at the indicated time points and quantified for (**A**) the quantity of infectious virions in conditioned media by TCID50 in Vero E6 cells and (**B–C**) SARS-CoV-2 RdRP copy number in RNA isolated from (**B**)conditioned media and (**C**) cellular lysates. (**D**) Calu-3 cells treated with nontargeting or *IFNAR1*-targeting gRNAs were infected with a SARS-CoV-2 nanoluciferase reporter strain, and the resulting luminescence signal was assayed at various time points post-infection. (**B–D**) Error bars represent standard deviations of four to six replicates for each assay, and asterisks indicate *P* < 0.05 by Student’s *t*-test.

The modifying influence of a separate or shared environment for *IFNAR1*-disrupted cells with control cells suggested a complexity of interactions with the extracellular space during SARS-CoV-2 infection. We reasoned that if *IFNAR1* disruption led to both a fitness-promoting cell autonomous effect and a fitness-reducing paracrine effect, then only the former would be isolated in a co-culture experiment in which both cell types shared the same media. When cultured separately, however, the impact on cell fitness would reflect the net effect of both signals. We therefore tested how endogenous *IFNAR1* disruption affected the extracellular space during SARS-CoV-2 infection. We found that conditioned media from *IFNAR1*-disrupted cells contained greater numbers of infectious virions, with both a higher peak at day 2 post-infection and a longer persistence at subsequent time points (Fig. 3A). Consistent with these findings, we also observed for *IFNAR1*-disrupted cells an increase in SARS-CoV-2 RNA associated with both the conditioned media (Fig. 3B) and cell monolayer (Fig. 3C), as well as an increase in luminescence during infection with a SARS-CoV-2 nanoluciferase reporter virus (42) (Fig. 3D). These findings support the importance of the endogenous IFN-I response in restricting SARS-CoV-2 replication in Calu-3 cells and suggest that in single cultures, any fitness-promoting effect conferred by IFN-I signaling disruption may be offset by an increase in viral replication.

### Persistent IFN-I signaling during SARS-CoV-2 infection limits the expansion of Calu-3 cells that survive early cytopathic effect

**Fig 4:**
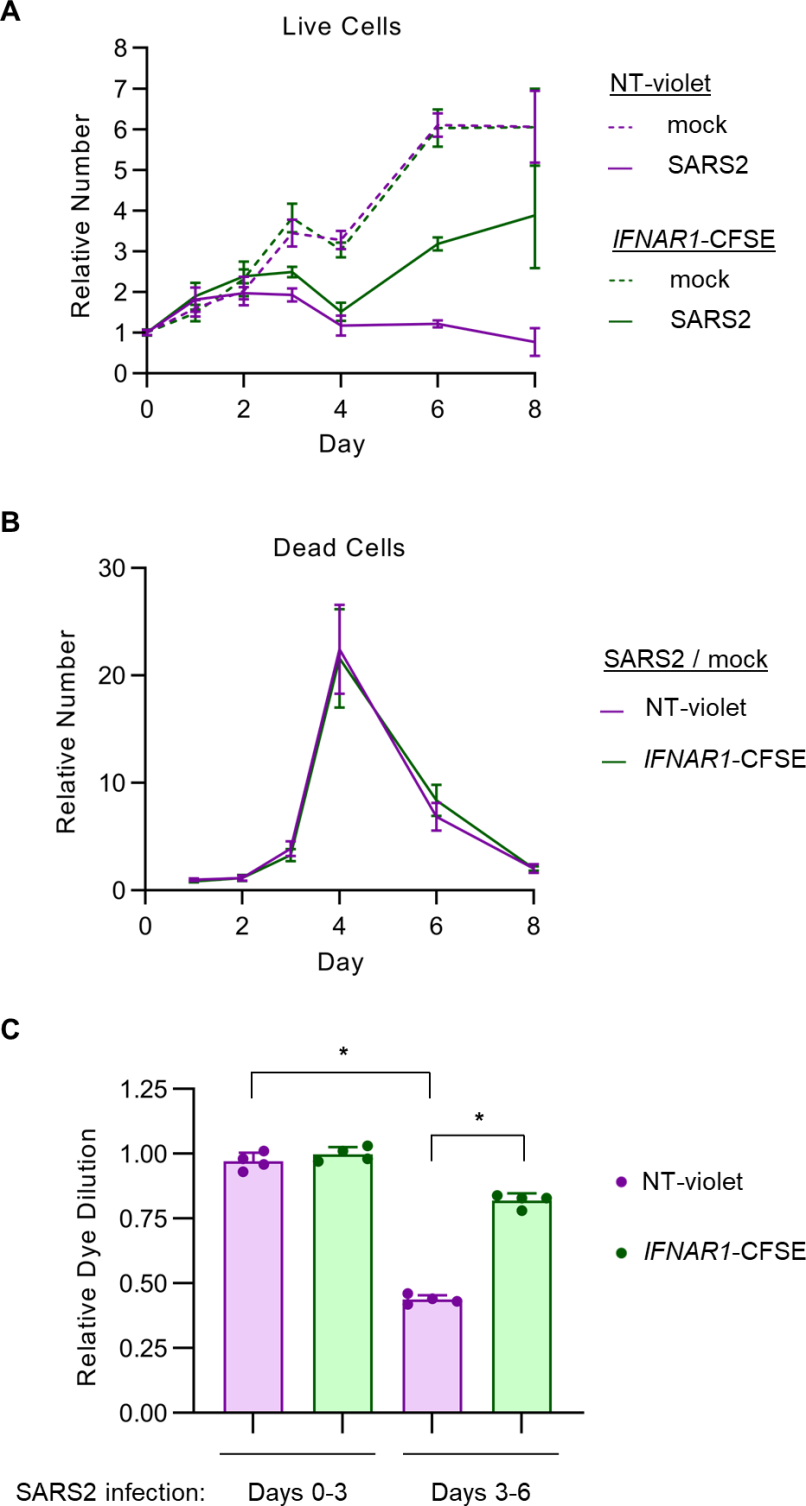
Persistent IFN-I signaling induces a delayed antiproliferative effect in SARS-CoV-2-infected Calu-3 cells. (**A–B**) Calu-3 cells were treated with a control nontargeting or *IFNAR1*-targeting gRNA and loaded with CellTrace-Violet or CellTrace-CFSE, respectively, pooled together, and either infected with SARS-CoV-2 WA1 strain at MOI of 1 (as derived from titering in Vero E6 cells) or mock-infected. The absolute number of cells for each population within the co-culture was calculated at each time point by flow cytometry with a comparison of event frequency to standard beads spiked into each sample at a known density. Live and dead cells were discriminated by staining with LIVE/DEAD Fixable Far Red viability dye. (**A**) Live cells adherent in the cell monolayer were normalized for each population to the beginning quantity of viable cells at day 0. (**B**) Dead cells detached in the conditioned media are plotted for SARS-CoV-2-infected conditions relative to mock-infected conditions at the same time point. (**C**) The proliferation of each cell population within the co-culture was quantified by the relative dilution of fluorescent dye over the indicated time interval, normalized to the proliferation of that cell type over the first 3 days of mock infection. Error bars represent standard deviations of four independent infections, and asterisks indicate *P* < 0.001 by Student’s *t*-test.

To better understand the basis for the fitness-decreasing effect of IFN-I signaling during SARS-CoV-2 infection, we next performed a detailed analysis of co-cultured control and *IFNAR1*-disrupted cells over the course of infection. We calculated absolute cell numbers by normalization to the event frequency of flow cytometry beads spiked into samples at a known concentration. Both control and *IFNAR1*-disrupted cells increased in number over the first 2 days of infection before reaching a plateau and then decreasing by day 4 post-infection (Fig. 4A), corresponding to the visual appearance of cytopathic effect in these cells. Subsequently, the number of control cells remained low throughout the remainder of the experiment until decay in the fluorescent signal precluded analysis beyond day 8. *IFNAR1*-disrupted cells, by contrast, underwent a robust increase in cell numbers from day 4 until day 8.

The delayed fitness advantage of *IFNAR1*-disrupted cells relative to control cells might have been caused by a decrease in cell death and/or an increase in cell proliferation. To distinguish between these possibilities, we tracked the absolute number of dead cells in either population over the course of SARS-CoV-2 infection. For each population, we observed far fewer dead cells associated with the cell monolayer than in the conditioned media (Fig. S7), suggesting that cell death led to rapid detachment of these cells from the plate. Relative to mock-infected conditions, the number of dead cells in the media rose abruptly on day 3 and peaked on day 4 post-infection (Fig. 4B), corresponding to the timing of the visual appearance of cytopathic effect and the decrease in viable cells associated with the monolayer (Fig. 4A). Compared to co-cultured control cells, *IFNAR1*-disrupted cells were equally sensitive to SARS-CoV-2-associated cell death (Fig. 4B).

We next monitored the cellular proliferation of control and *IFNAR1*-disrupted cells over time by measuring the dilution of either fluorescent dye with each cell doubling. Both cell types exhibited comparable rates of proliferation over the first 3 days of either mock or SARS-CoV-2 infection (Fig. 4C). Between days 3 and 6, however, SARS-CoV-2-infected control cells exhibited a clear decrease in dye dilution (Fig. 4C), corresponding to their lack of proliferation during this time (Fig. 4A). *IFNAR1*-disrupted cells, by contrast, were resistant to this decrease in dye dilution between days 3 and 6 post-infection (Fig. 4C), consistent with the expansion of this population during this time (Fig. 4A). Together, these findings indicate that persistent IFN-I signaling during SARS-CoV-2 infection reduces the fitness of Calu-3 cells through a cell autonomous antiproliferative response rather than a sensitization to cell death.

### Analysis of factors influencing the fitness advantage of *IFNAR1*-disrupted Calu-3 cells during SARS-CoV-2 infection

Recently, three other studies have reported high-throughput CRISPR screens for modifiers of Calu-3 cell fitness during SARS-CoV-2 infection (31–33). We compared the genes identified in our screen with the findings from each study (Fig. S8). Although *ACE2* was a top hit in each screen, and fitness-promoting and fitness-reducing gene perturbations were broadly correlated between the studies, there was otherwise a modest overlap between the results of our screen and these studies. Like us, Biering et al. detected a significant association between disruption of *IFNAR1* and *IFNAR2* with increased Calu-3 cell fitness during SARS-CoV-2 infection, though with a magnitude of enrichment less than we observed (32). Rebendenne et al. did not detect *IFNAR1*, *IFNAR2*, *JAK1*, or *TYK2* among the top hits of their primary genome-wide screen, though interestingly they did identify *IRF9*, which is downstream of canonical IFN-I signaling, as the top hit of their secondary screen despite this gene showing no significant effect in the primary genome-wide screen (33). *IRF9* was also identified by Biering et al., along with the other genes encoding its trimeric transcription factor complex*—STAT1* and *STAT2* (32). Israeli et al. did not detect a significant effect for disruption of IFN-I signaling during SARS-CoV-2 infection of Calu-3 cells (31). Each of these studies had technical differences in experimental design, including the multiplicity of infection, the plating density of cells, and the duration of culturing after infection before gRNA analysis.

**Fig 5:**
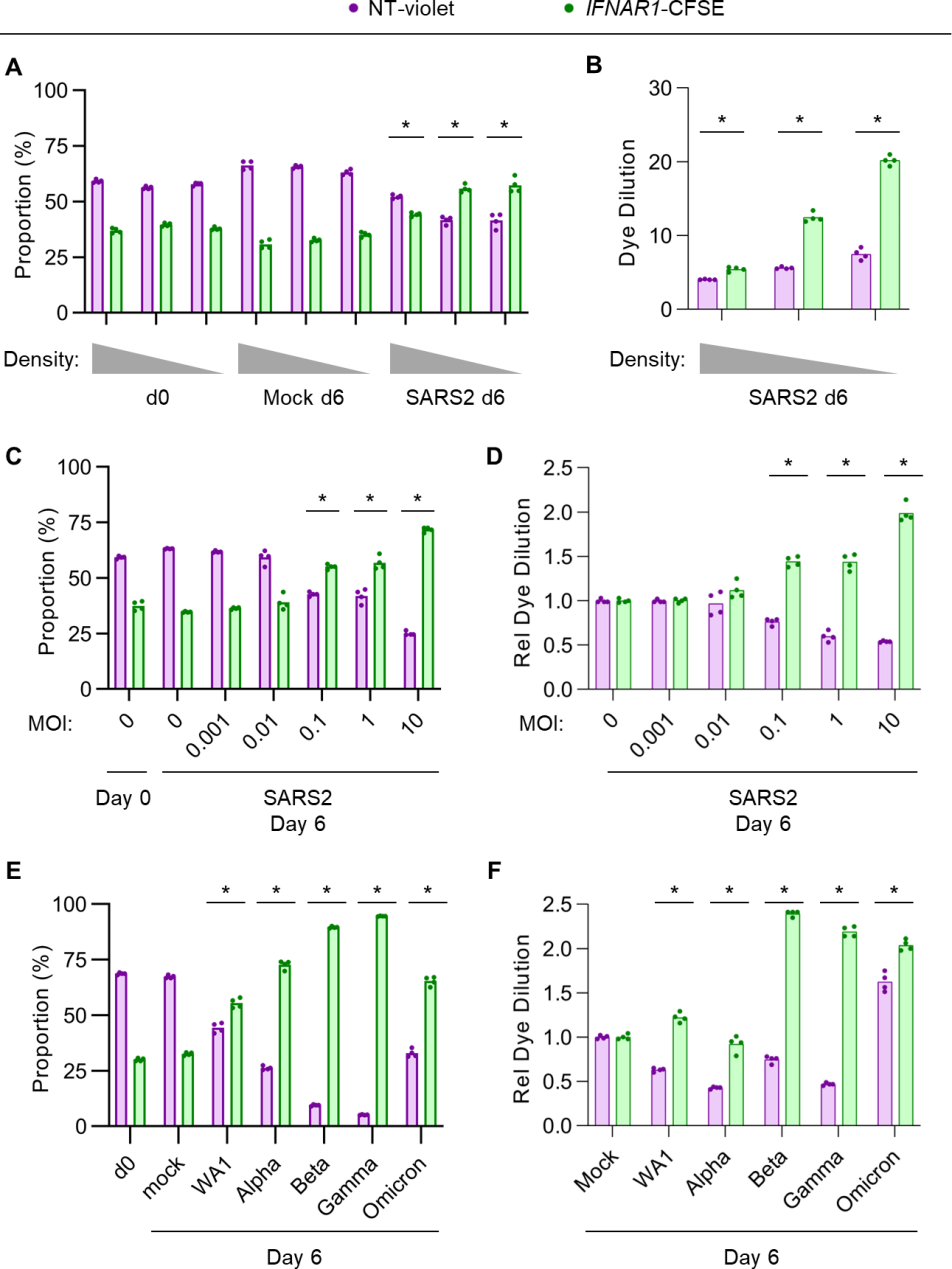
The competitive advantage conferred by IFNAR signaling disruption during SARS-CoV-2 infection is modulated by seeding density and viral MOI. Co-cultures of nontargeting gRNA-treated, CellTrace-Violet-loaded Calu-3 cells and *IFNAR1*-targeting gRNA-treated, CellTrace-CFSE-loaded cells were (**A–B**) seeded at a range of plating densities and infected with SARS-CoV-2 WA1 strain at MOI of 1 or (**C–F**) seeded at the same ~20% plating density and infected with (**C–D**) SARS-CoV-2 WA1 strain at a range of MOI or (**E–F**) SARS-CoV-2 variants at MOI of 1. The relative proportion of each cell population was analyzed by flow cytometry at the indicated time points (**A, C, E**), and the dilution of either fluorescent dye was quantified by changes in mean fluorescence intensity, normalized to the dilution of either dye under mock-infected conditions. All MOI estimates were derived from titering experiments on Vero E6 cells. Individual data points represent independent infections, and asterisks indicate *P* < 0.01 by Student’s *t*-test between SARS-CoV-2 and mock-infected conditions (**A, C, E**) or between nontargeting and *IFNAR1*-targeting gRNA (**B, D, F**).

To analyze how different experimental parameters might influence the competitive advantage of IFN-I signaling disruption during Calu-3 SARS-CoV-2 infection, we again tracked the relative abundance of co-cultured control and *IFNAR1*-disrupted cells under different conditions. We found that lower plating densities of Calu-3 cells led to a greater competitive advantage for *IFNAR1*-disrupted cells (Fig. 5A), associated with a greater degree of proliferation for these cells (Fig. 5B). The amount of virus used for infection also had a large modifying influence, with increasing MOI leading to a more pronounced proliferation advantage for *IFNAR1*-disrupted cells (Fig. 5C and D). The competitive advantage of *IFNAR1*-disrupted cells was not unique to the WA1 strain, as it was also observed for each of the Alpha, Beta, Gamma, and Omicron variants of SARS-CoV-2, albeit with varying magnitude of effect (Fig. 5E and F). These findings suggest that experimental conditions that are associated with more cell doublings after the initial cytopathic effect and greater production of IFN-I are more likely to reveal a proliferation advantage conferring by IFN-I signaling disruption.

### IFN-I signaling alone, in the absence of viral infection, is sufficient to induce an antiproliferative response in Calu-3 cells

**Fig 6:**
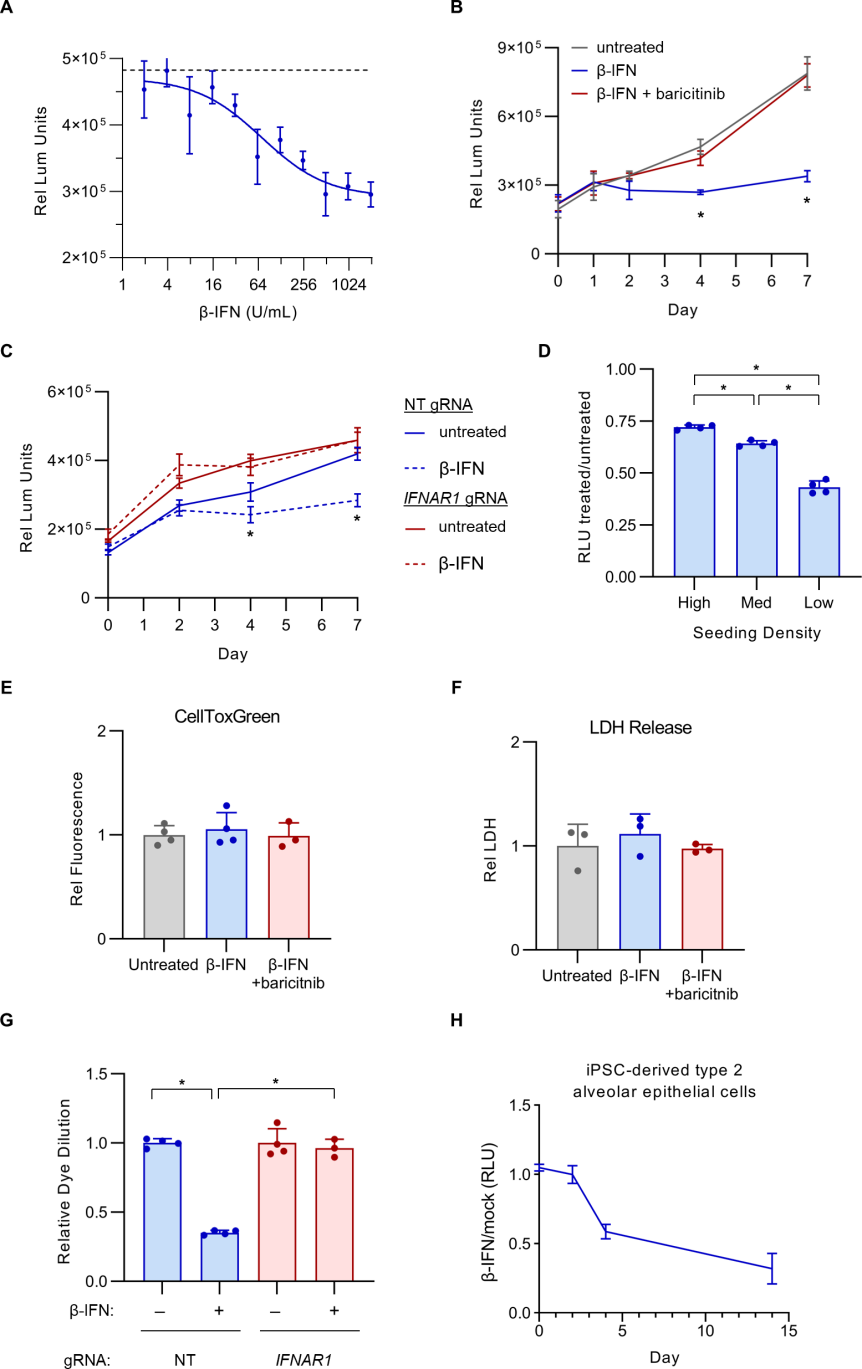
Exogenous IFN-I alone, in the absence of viral infection, is sufficient to induce an antiproliferative response in Calu-3 cells. (**A**) Calu-3 cells were incubated with a range of concentrations of exogenous β-IFN, and the effect on cell quantities after 4 days was measured by CellTiter-Glo luminescence. (**B**) Calu-3 cells were incubated in the presence or absence of 1,000 U/mL β-IFN with or without 1 µM baricitinib and analyzed by CellTiter-Glo luminescence at the indicated time points. (**C**) Calu-3 cells treated with a control NT gRNA or a *IFNAR1*-targeting gRNA were incubated in the presence or absence of 1,000 U/mL β-IFN and analyzed by CellTiter-Glo luminescence at the indicated time points. (**D**) Calu-3 cells were seeded at a range of densities (estimated confluence high = 80%, medium = 40%, and low = 20%), incubated with or without 1,000 U/mL β-IFN, and analyzed by CellTiter-Glo luminescence. The relative luminescence units of treated samples relative to untreated samples are plotted for each plating density. (**E–F**) Untreated and β-IFN-treated cells were analyzed for the presence of cell death after 4 days, as assayed by (**E**) cellular exclusion of the fluorescent viability dye CellToxGreen or (**F**) quantification of LDH released to the conditioned media. (**G**) The relative proliferation of untreated and β-IFN-treated Calu-3 cells was compared by flow cytometry detection of CellTrace-CFSE dilution over time. Error bars indicated standard deviations for technical replicates, and asterisks indicate *P* < 0.001 by Student’s *t*-test. (**H**) Type 2 alveolar epithelial cells were differentiated from iSCP cells, seeded into multiwell plates, and monitored for proliferation by CellTiter-Glo luminescence at serial time points after treatment with 1,000 U/mL exogenous β-IFN or vehicle. The ratio of luminescence for β-IFN-treated versus vehicle-treated cells over time is displayed.

The resistance of *IFNAR1*-disrupted cells to the delayed antiproliferative response during SARS-CoV-2 infection indicated that IFN-I signaling was necessary for this response. To test whether it was also sufficient, we next treated uninfected Calu-3 cells with exogenous β-IFN at a range of concentrations and measured the impact on cell viability and proliferation. We observed a dose-dependent decrease in viable cells upon β-IFN treatment (Fig. 6A), with an estimated IC_50_ that was approximately 20-fold lower than the reported concentration of endogenous β-IFN for wild-type Calu-3 cells at the peak of SARS-CoV-2 infection (34). A time course analysis revealed the onset of this effect between days 2 and 4 post-treatment (Fig. 6B), similar to the kinetics observed during SARS-CoV-2 infection (Fig. 4A). This effect was dependent on IFN-I signaling, as it was prevented by either treatment with baricitinib (Fig. 6B) or genetic ablation of *IFNAR1* (Fig. 6C). Also consistent with our findings during SARS-CoV-2 infection (Fig. 5A and B), the magnitude of the antiproliferative response was dependent on cell density, as Calu-3 cells plated at lower density exhibited a greater decrease in viable cells upon β-IFN treatment compared to cells plated at higher densities (Fig. 6D). Likewise, the β-IFN-mediated decrease in viable cells was not associated with an increase in cell death, as measured by membrane exclusion of a viability dye (Fig. 6E) or by LDH release to the extracellular environment (Fig. 6F), but rather by a reduction in cellular proliferation, as measured by the dilution of fluorescent dye over time (Fig. 6G).

Different cell types that exhibit an antiproliferative response to IFN-I treatment have been found to vary in the specific stage(s) at which cell cycle progression is delayed (43–46). We performed propidium iodide staining and flow cytometry of untreated and β-IFN-treated Calu-3 cells, finding no significant shift in cell cycle distribution over 4 days of treatment (Fig. S9). These findings likely reflect the simultaneous inhibition of multiple stages of cell cycle progression, as has been demonstrated for a subset of cell types with IFN-I-sensitive proliferation (43, 47, 48).

To examine the generalizability of the Calu-3 IFN-I antiproliferative response in a nontransformed model of respiratory epithelial cell biology, we also generated type 2 alveolar epithelial cells from human-induced pluripotent stem cells. Treatment of these cells with exogenous β-IFN treatment resulted in a reduction in cell fitness of a comparable magnitude and timing as we had observed for Calu-3 cells (Fig. 6H). Taken together, these findings indicate that IFN-I signaling is both necessary and sufficient to induce an antiproliferative effect in respiratory epithelial cells during SARS-CoV-2 infection and suggest that this response may be generalizable to other pathologic states associated with persistent IFN-I signaling in the lung epithelium.

## DISCUSSION

Because of its central importance in host defense against a wide variety of viral pathogens, the IFN-I response to COVID-19 has been the subject of extensive investigation since the onset of the pandemic. Several studies have found that pre-treatment of cultured cells with type I interferons before SARS-CoV-2 infection leads to a clear reduction in viral replication (49–53), and this effect is mirrored by limited clinical trial data suggesting a benefit to patients for treatment with exogenous type I interferon prior to the onset of symptoms (54). Human genetic studies have also suggested a link between interferon responses and COVID-19 outcomes, with one group finding 3.5% of patients with severe COVID-19 to harbor rare variants in interferon signaling genes (including *IFNAR1*, *IFNAR2*, *IRF3*, and *IRF7*) (55), though this finding was not replicated in other studies (56, 57). Autoantibody profiling of patients with severe COVID-19 also detected neutralizing antibodies against circulating interferons in 10.2% of patients (58). Our finding that genetic or pharmacologic inhibition of IFN-I signaling leads to an increase in SARS-CoV-2 replication in Calu-3 cells is consistent with these studies demonstrating a protective effect for early IFN-I signaling during SARS-CoV-2 infection.

Despite the potential benefits of early IFN-I signaling during SARS-CoV-2 infection, several lines of evidence indicate that its persistent activation contributes to the progression of lung disease in severe COVID-19. Longitudinal profiling of patients with COVID-19 has revealed a correlation between persistent IFN-I responses and greater severity of disease (59–64). In mouse models of SARS-CoV-2 infection, genetic ablation of *Ifnar1* (18) and pharmacologic inhibition of IFN-I production (19) have each been shown to protect against lung pathology, while the administration of exogenous IFN-I after the onset of symptoms in humans and mice has been found to worsen disease severity (20, 65, 66). A similar pathogenic effect for persistent IFN-I signaling has also been demonstrated in mouse models of SARS-CoV-1 (67) and MERS-CoV (68) infection. Although specific IFN-I signaling inhibitors have not been tested in clinical trials, the Janus kinase inhibitors baricitinib and tofacitinib, which block IFN-I signaling (in addition to other pathways), have been shown to improve outcomes in severe COVID-19 (14–16). Together, these studies all support the overall pathogenic effect of persistent IFN-I signaling at advanced stages of SARS-CoV-2 infection but do not identify the cell type or downstream pathways responsible for this effect.

Unexpectedly, our unbiased CRISPR screening approach revealed a cell autonomous and detrimental effect of persistent IFN-I signaling on Calu-3 cells during SARS-CoV-2 infection. The competitive advantage conferred by IFN-I signaling disruption in our screen might have been mediated by either protection from SARS-CoV-2-induced cell death or increase in cellular proliferation, and IFN-I signaling has been implicated in both processes in different cell types (45, 69). We however found no influence for IFN-I signaling on Calu-3 cell death, with *IFNAR1*-disrupted cells showing equal sensitivity to SARS-CoV-2-induced death (Fig. 4B) and exogenous IFN-I treatment causing no increase in cell death (Fig. 6E and F). Instead, we found that the fitness advantage of *IFNAR1*-disrupted cells during SARS-COV-2 infection was mediated by an increase in cell proliferation between days 4 and 8 post-infection (Fig. 4A and C), a finding that was mirrored by the antiproliferative response of uninfected Calu-3 and iPSC-derived type 2 alveolar epithelial cells to exogenous β-IFN treatment (Fig. 6G and H).

Other high-throughput CRISPR screens for host factors affecting SARS-CoV-2 infection have not identified the same magnitude of protection as we observed for IFN-I signaling disruption. In some cases, this is likely due to the cell type tested. Vero E6 cells, for example, are deficient in the endogenous production of type I interferons (70). Even for other screens of Calu-3 cells (31–33), however, the observed effect for IFN-I signaling disruption has been variable (Fig. S8). This is likely related to differences in screen design, as we identified multiple factors that influenced the relative advantage conferred by IFN-I signaling disruption. For example, Biering et al. harvested cells at an earlier time point (day 5 post-infection rather than day 14 in our screen) (32), which likely allowed for the better detection of gene disruptions that affected cell death rather than the outgrowth of surviving cells. We likewise found that the magnitude of competitive advantage conferred by IFN-I signaling disruption was influenced by the plating density of the Calu-3 cells, the MOI of SARS-CoV-2 infection, and the SARS-CoV-2 variant tested. These discrepancies highlight how the technical nuances of screen design may be biased toward the detection of host genes acting at different stages of viral infection. Additionally, our screen involved deeper coverage for a more limited set of candidate genes, likely increasing our power to resolve subtle differences in the effect sizes of gene disruption.

The potential of IFN-I signaling to induce an antiproliferative response has long been recognized (71, 72), but the sensitivity of different cell types to these (and other) IFN-I responses varies considerably (43–46). Our findings suggest that respiratory epithelial cells are highly sensitive to the antiproliferative effect of IFN-I signaling and that during SARS-CoV-2 infection, this response has a greater impact on cell fitness than any concurrent activation of antiviral or regulated cell death pathways. Several possible mechanisms for IFN-I antiproliferative activity at different stages of cell cycle progression have been identified in other cell types. These include upregulation of cyclin-dependent kinase inhibitors (73–75), downregulation of the transcription factor c-myc (76, 77), activation of the tumor suppressors Rb (78, 79) and p53 (80, 81), and crosstalk with other signaling pathways (82, 83). Our observation that IFN-I signaling does not alter the overall cell cycle distribution of Calu-3 cells implies that multiple effectors are likely involved, similar to what has been proposed for other cell types that exhibit an antiproliferative response to IFN-I without arrest at a particular cell cycle stage (43).

*In vivo*, the proliferation of respiratory epithelial cells is known to play a crucial role in recovery from acute lung injury, as progenitor cells (especially AT2 cells) generate a pool of cells that repopulate the injured alveolar lining (84). The efficiency of this response is tightly linked to the clinical outcome of patients with acute respiratory distress syndrome (85–87). Intriguingly, IFN-I signaling has been shown to negatively regulate AT2 proliferation in a mouse model of influenza (88). It is unclear whether this interaction between IFN-I signaling and AT2 proliferation is unique to influenza or extends to other IFN-I-associated causes of acute lung injury. Our finding that IFN-I signaling alone is sufficient to cause an antiproliferative response in respiratory epithelial cells supports the potential generalizability of this effect. Our findings also suggest that the therapeutic benefit of Janus kinase inhibitors in severe COVID-19 may be due to their protection against an IFN-I-mediated antiproliferative response in respiratory epithelial cells. Future investigations will be necessary to establish the significance of these findings *in vivo* and to explore the therapeutic potential of specifically targeting the IFN-I-mediated antiproliferative response during SARS-CoV-2 infection and other IFN-I-associated causes of acute lung injury and ARDS.

## MATERIALS AND METHODS

### Cell lines and virus stocks

Calu-3 and HEK-293T cells were obtained from ATCC (Manassas, VA) and cultured in Dulbecco’s modified Eagle’s medium (DMEM) supplemented with 10% fetal bovine serum (FBS), 10 U/mL penicillin, and 10 µg/mL streptomycin in a humidified 5% CO_2_ chamber at 37°C. Cell lines were periodically tested for mycoplasma contamination and identity confirmation by microsatellite genotyping. SARS-CoV-2 WA1 (isolate USA-WA1/2020, lineage A, catalog NR-52281), Alpha (isolate USA/CA_CDC_5574/2020, lineage B.1.1.7, catalog NR-54011), Beta (isolate hCoV-19/USA/MD-HP01542/2021, lineage B.1.351, catalog NR-55282), Gamma (isolate hCoV-19/Japan/TY7-503/2021, lineage P.1, catalog NR-54982), and Omicron (isolate hCoV-19/USA/MD-HP20874/2021, lineage B.1.1.529, catalog NR-56461) variants were obtained from BEI Resources and propagated in Vero E6 cells. SARS-CoV-2 encoding nanoluciferase (42) was kindly provided by Dr. R. Baric (University of North Carolina). The lack of genetic drift in viral stocks was confirmed by full-length sequencing as previously described (89). Viral titers were determined by TCID50 assays in Vero E6 cells by microscopic scoring using the Reed–Muench method (90). All experiments using SARS-CoV-2 were performed at the University of Michigan under Biosafety Level 3 protocols in compliance with containment procedures in laboratories approved for use by the University of Michigan Institutional Biosafety Committee and Environment, Health and Safety.

### CRISPR screen design

The design and synthesis of the custom CRISPR library were previously described (35). For every two independent biologic replicates, a total of ~50 million Calu-3 cells were transduced with the lentiviral CRISPR library at an MOI of ~0.3. Puromycin was added at a concentration of 3 µg/mL at day 1 post-transduction and maintained until the selection of control uninfected cells was complete. Cells were passaged as needed to maintain the logarithmic phase growth with the total cell number maintained above a minimum of 10 million cells at all stages of the screen. At 14 days post-transduction, a total of ~40 million cells in four T175 flasks at approximately 50% density were infected with SARS-CoV-2 WA1 strain in 10 mL infection media (DMEM with 2% FBS). The amount of virus for each infection was calculated to correspond to an MOI of 1.0 based on titering in Vero E6 cells; the proportion of SARS-CoV-2-infected cells at 48 h was also assessed by immunofluorescence of SARS-CoV-2 nucleocapsid protein, as previously described (91, 92). Two days post-infection, with the incursion of visual cytopathic effect, 15 mL of maintenance media (DMEM with 10% FBS) was added. Cells were inspected every 2 days, and the medium was replaced at day 6 and 10 post-infection to allow the enrichment of surviving cells. Cells were harvested at day 14 post-infection, genomic DNA was extracted with the QIAamp DNA Mini Kit, and gRNA sequences were amplified and sequenced as previously described (93).

### CRISPR screen analysis

FASTQ files were processed by PoolQ (Broad Institute; https://portals.broadinstitute.org/gpp/public/software/poolq) to map individual sequencing reads to reference gRNA sequences with deconvolution of sample identity by barcode. Cumulative distribution functions of gRNA representation were generated by plotting normalized read counts of each gRNA against its relative rank for a given barcode. Individual gRNA-level and aggregate gene-level enrichment analysis was performed using MAGeCK (39). *Q*–*Q* plots were generated by plotting log-transformed observed *P*-values (calculated by MAGeCK gene-level analysis) against expected *P*-values (determined by the relative rank of each gene in the library). Genes were considered screen hits if they were identified with a MAGeCK-calculated false discovery rate <0.05. Identified genes were filtered to remove those genes for which disruption influenced the fitness of uninfected cells, as reflected by a significant (FDR < 5%) aggregate enrichment (log2FC > 1, 14 genes) or depletion (log2FC<−1, 108 genes) of gRNAs prior to SARS-CoV-2 infection, relative to the plasmid pool. Genes targeted by depleted gRNAs were compared to the mean gene effect score in CRISPR screens of 1,070 cell lines in the Broad Institute Cancer Dependency Map release 22Q1 (40). Enrichment of functional annotations among screen hits relative to all genes in the CRISPR library was performed using GOrilla with a default setting of *P*-value <10^−3^ for Gene Ontology Biologic Processes (94); when multiple nodes were identified within the same hierarchy, the most significantly enriched term was selected for visualization. Gene network analysis of screen hits was performed using the STRING database (95) with default settings and visualized using Cytoscape (96) v3.9.1 with node borders weighed by −log(RRA score) for each gene in the CRISPR screen and edges weighed by the gene–gene interaction score in the STRING database. Heatmaps were generated with GraphPad Prism v9.1.0 from published CRISPR screen data, using each gene’s aggregate gRNA log2-fold change or *Z*-score and the limits of the color spectrum assigned to values at the 99.9 and 0.1 percentiles within each screen.

### Quantification of Calu-3 SARS-CoV-2 infection

Calu-3 cell infectivity by SARS-CoV-2 was determined by a previously developed high-content imaging assay (97). Briefly, Calu-3 cells were cultured on 384-well phenoplates, allowed to adhere overnight, and either mock-infected or infected with SARS-CoV-2. Cells were then incubated for 48 h at 37°C, fixed with 5% paraformaldehyde, permeabilized with 0.3% Triton X-100, and stained for nuclei, cell region, and nucleocapsid protein. Images were acquired with a Yokogawa CQ-1 High Content System. Cell segmentation and feature extraction were done with CellProfiler. Cells were determined to be infected if the fluorescence intensity of the nucleocapsid was greater than 1.96 standard deviations above the mean.

### Generation of *IFNAR1*-disrupted and control nontargeting cells

CRISPR lentiviral stocks were generated by ligating a *IFNAR1*-targeting (GTACATTGTATAAAGACCAC) or control nontargeting sequence (GTTCATTTCCAAGTCCGCTG) into *BsmBI*-digested pLentiCRISPRv2 (98), co-transfecting each construct with psPAX2 and pVSVG into HEK293T cells, harvesting supernatants, and titering virus stocks as previously described (99). Calu-3 cells were transduced in parallel with each lentiviral construct, treated with puromycin 3 µg/mL until no surviving cells remained among control nontransduced cells, and passaged to retain the logarithmic phase growth. Phenotypic analysis was performed after a minimum of 14 days post-transduction to ensure adequate time for target site editing and turnover of residual protein.

### Analysis of cells with activation or inhibition of IFN-I signaling

Calu-3 cells were treated with exogenous β-IFN (R&D Systems, Minneapolis, MN), baricitinib (Selleck Chemicals, Houston, TX), or vehicle at the indicated time points and concentrations. Total RNA was isolated from cell lysates or the conditioned media using the RNeasy Plus Micro kit (Qiagen, Hilden, Germany) or QIAamp Viral RNA kit (Qiagen), respectively, and converted to cDNA with the SuperScript III First-Strand Synthesis kit (Thermo Fisher, Waltham, MA). Individual transcripts were amplified with primer pairs for *IFNAR1* (proprietary sequence, assay number Hs.PT.58.20048943, Integrated DNA Technologies, Coralville, IA), *IFIT1* (AAGCTTGAGCCTCCTTGGGTTCGT and TCAAAGTCAGCAGCCAGTCTCAGG), *IFITM1* (CCAAGGTCCACCGTGATTAAC and ACCAGTTCAAGAAGAGGGTGTT), and SARS-CoV-2 RdRP (F2 primer IDT catalog 10006860 and R1 primer IDT catalog 1000688) using the Power SYBR Green PCR Master Mix (Thermo Fisher), and analyzed by QuantStudio 5 Real-Time PCR (Thermo Fisher). The quantification of individual transcripts was normalized against a panel of *ACTB*, *RPL37*, and *RL38* loading controls, as previously described (100). Immunoblotting of RIPA lysates was performed as previously described (101) with antibodies against IFNAR1 (Abcam, Cambridge, UK, ab124764, 1:500), IFITM3 (Proteintech, Rosemont, IL, 117141, 1:500), and β-actin (Santa Cruz Biotechnology, Dallas, TX, sc-47778, 1:5000). The quantity of viable cells over time was monitored using CellTiter-Glo 2.0 (Promega, Madison, WI) according to the manufacturer’s instructions, with luminescence read on a VICTOR Nivo plate reader (PerkinElmer, Waltham, MA). Cell death was monitored using the LDH-Glo Cytotoxicity Assay (Promega), CellToxGreen (Promega), or flow cytometry of cells stained with LIVE/DEAD Fixable Far Red viability dye (Thermo Fisher). For cell cycle analysis, Calu-3 cells were pre-treated with 1,000 U/mL exogenous β-IFN (R&D) or vehicle for 1, 2, 3, or 4 days, harvested, stained with PI/RNase Staining Solution (Thermo Fisher) as per the manufacturer’s instructions, and analyzed by flow cytometry.

### Competitive co-culture experiments

Control and *IFNAR1*-disrupted Calu-3 cells were detached using TrypLE Express, collected in DMEM with 10% FBS, and centrifuged at 500 × *g* for 5 min, and the supernatant was aspirated. Cell pellets were then resuspended in 8 mL PBS pre-warmed at 37°C and CellTrace-Violet or CellTrace-CFSE added to the suspension to a final concentration of 5 µM. Cell suspensions were then incubated at 37°C for 20 min with periodic gentle agitation. Labeling was then quenched by the addition of 40 mL DMEM with 10% FBS at 4°C and cells centrifuged as above. The supernatant was aspirated, and cell pellets were resuspended in DMEM with 10% FBS. Cells were then pooled together and seeded into six-well plates at a confluence of approximately 20% or as otherwise indicated. SARS-CoV-2 infections were performed 4 days later as described above. Media were replaced the day after seeding the cells, the day prior to SARS-CoV-2 infection, and approximately every 3–4 days after infection. At the indicated time points, cells were detached, washed, stained with LIVE/DEAD Fixable Far Red viability dye (Thermo Fisher) for 15 min at 4°C, washed, fixed with 4% paraformaldehyde for 15 min at room temperature, and analyzed by flow cytometry. Where indicated, CountBright Absolute Counting Beads (Thermo Fisher) were spiked into samples at 1,000 beads/µL immediately prior to flow cytometry. Flow cytometry was performed on a BioRad Ze5 instrument. Gates were established based on control populations of cells labeled with either individual dye and unlabeled cells that were cultured in isolation. Analysis of event gating frequency and mean fluorescence intensity was performed using FlowJo v10.8.1.

### Derivation and analysis of iPSC-derived AT2 cells

Type 2 alveolar epithelial cells were differentiated from induced pluripotent stem cells (SPC2 line, clone SPC2-ST-B2, Boston University) as previously described (102–104), including withdrawal of CHIR99021 on day 32 and addback on day 37. Fluorescence microscopy was used to confirm the expression of the *SFTPC*:tdTomato reporter gene. Differentiated cells were maintained in “CK+DCI” media as three-dimensional alveolospheres embedded in Matrigel (Corning Incorporated, Corning, NY). Passaging was performed approximately every 2 weeks by trypsin dissociation of single cells followed by embedding into fresh Matrigel at a density of 400 cells/µL or, for two-dimensional experiments, seeding into tissue culture–treated plates that were additionally coated with 67 µg/mL Human Collagen Type IV (Sigma) for 2 hours at 37°C. At passage or when seeding to 2D experiments, 10 µM Rho-associated kinase inhibitor (Y-27632) (Thermo Fisher) was added to media for the first 24 hours to increase cell survival. The proliferation of viable cells over the duration of treatment with 1,000 U/mL β-IFN (R&D) or PBS vehicle control was monitored by CellTiterGlo 2.0 luminescence (Promega), as described above for Calu-3 cells.

## Data Availability

Full CRISPR screen results are available in the supplemental tables.

## SUPPLEMENTAL MATERIAL

Supplemental figures jvi.01276-23-s0001.docx Figures S1 to S9 and legends for supplemental tables.

Table S1 jvi.01276-23-s0002.xlsx CRISPR screen gene level analysis, SARS-CoV-2 infection.

Table S2 jvi.01276-23-s0003.xlsx CRISPR screen gRNA level analysis, SARS-CoV-2 infection.

Table S3 jvi.01276-23-s0004.xlsx CRISPR screen gene level analysis, pre-infection.

Table S4 jvi.01276-23-s0005.xlsx CRISPR screen gRNA level analysis, pre-infection.

Table S5 jvi.01276-23-s0006.xlsx Raw data for statistical analyses.

ASM does not own the copyrights to Supplemental Material that may be linked to, or accessed through, an article. The authors have granted ASM a non-exclusive, world-wide license to publish the Supplemental Material files. Please contact the corresponding author directly for reuse.
