# Supplementary material for: Type I interferon signaling induces a delayed antiproliferative response in respiratory epithelial cells during SARS-CoV-2 infection
Source: J Virol. 2023 Nov 17;97(12):e01276-23. doi: 10.1128/jvi.01276-23 (PMC10734423; doi:10.1128/jvi.01276-23)
Supplement: Supplemental figures — Figures S1 to S9 and legends for supplemental tables. [file jvi.01276-23-s0001.docx]

**
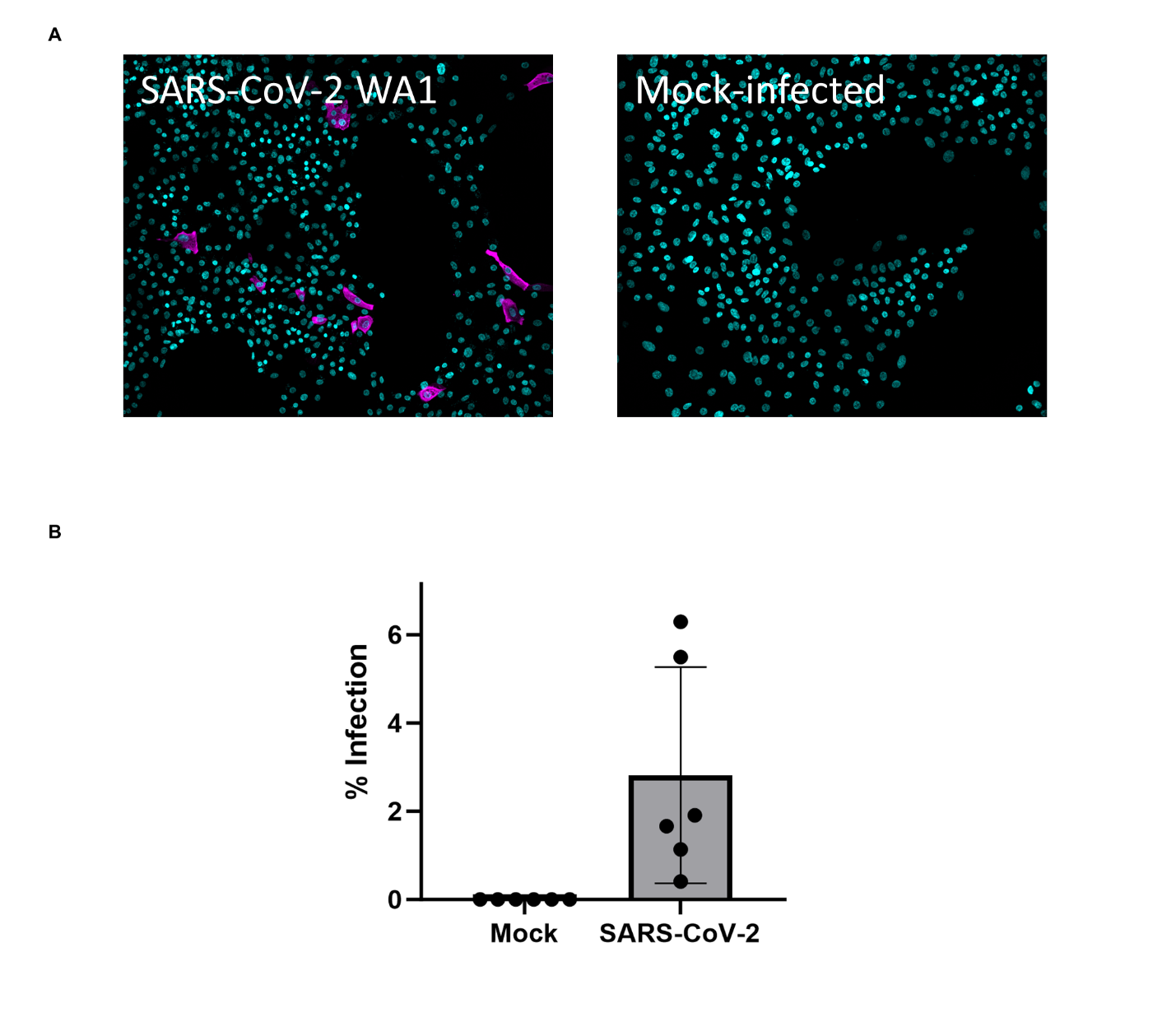
Figure S1. Immunofluorescence of SARS-CoV-2-infected Calu-3 cells.** (A) Calu-3 cells were mock-infected or infected with SARS-CoV-2 WA1 strain at an MOI of 1.0, based on calculations derived from titering in Vero E6 cells. Immunofluorescence was performed with staining of the SARS-CoV-2 nucleocapsid protein (purple) and counterstaining with DAPI (blue). (B) The proportion of infected cells under either condition was quantified by automated image analysis for each of 6 replicates.

**
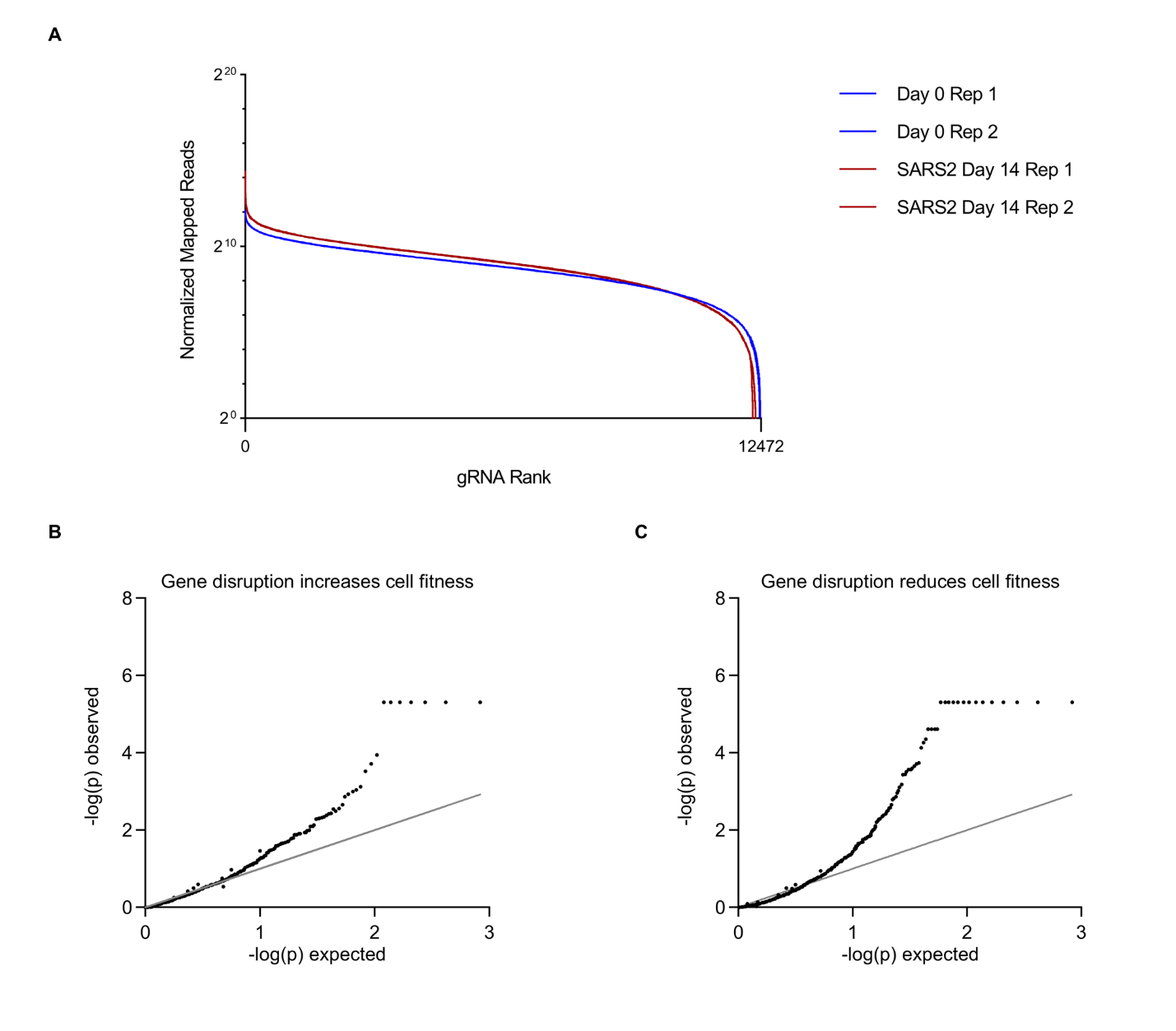
Figure S2. CRISPR screen analysis.** (A) Cumulative distribution functions of normalized read counts for each gRNA before and after 14 days of SARS-CoV-2 infection for each of 2 independent biologic replicates. (B) Q-Q plots of observed versus expected –log(p) for gene perturbations associated with increased (B) or decreased (C) cell fitness during SARS-CoV-2 infection for every gene targeted by the CRISPR library. Observed p-values were calculated by MAGeCK gene-level analysis (Supplemental Table 1).

**
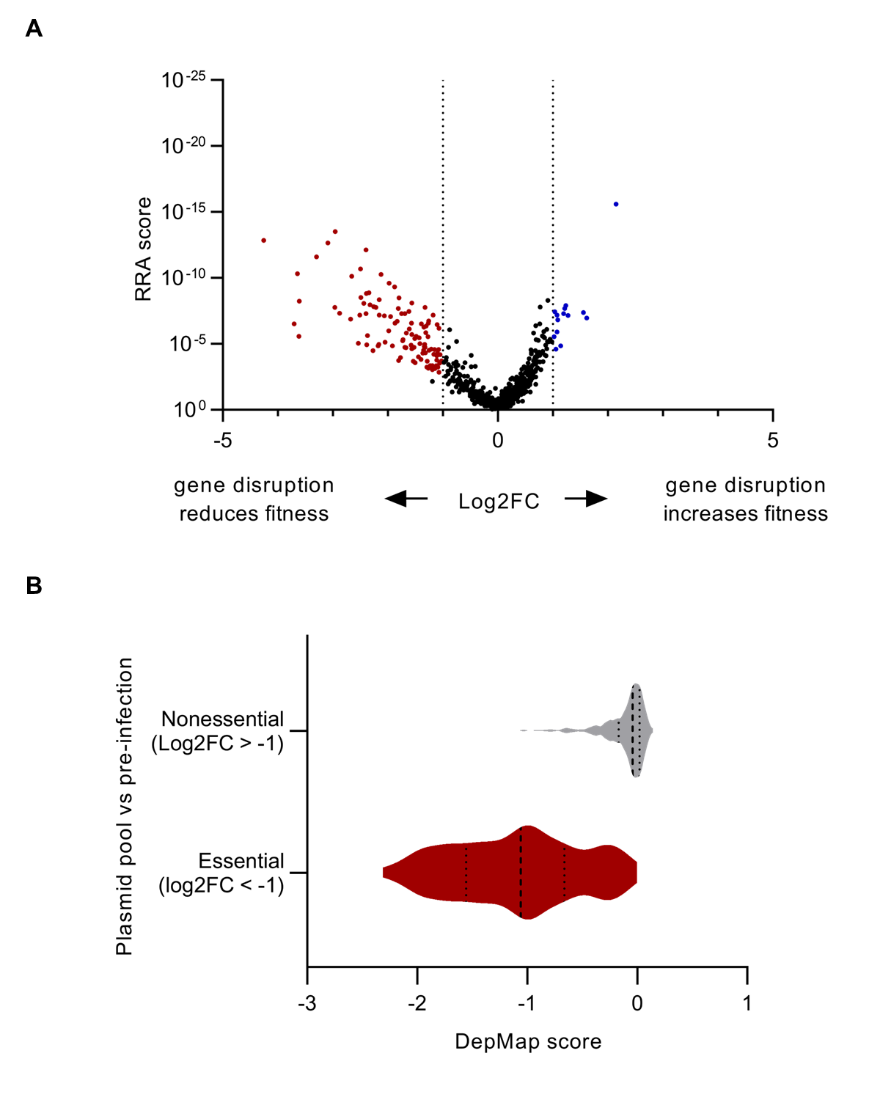
Figure S3. Filtering of genes with influence on Calu-3 cell fitness independently of SARS-Cov-2 infection.** (A) Volcano plot of gRNA enrichment in day 0 pre-infected cells relative to the CRISPR library plasmid pool. Genes whose disruption conferred a significant (FDR<5%) increase (aggregate log2FC>1) or decrease (aggregate log2FC <-1) in cell fitness are highlighted in blue or red, respectively, and were filtered out of the CRISPR screen results for modifiers of cell fitness during SARS-CoV-2 infection. Source data is provided in Supplemental Tables 3 and 4. (B) Aggregate DepMap essentiality scores derived from CRISPR screens of 1070 cell lines for genes identified in this study as nonessential (L2FC>-1) or essential (L2FC<-1, FDR<5%) in Calu-3 cells prior to the onset of SARS-CoV-2 infection. DepMap scores of 0 indicate neutral effect on cell fitness while significant negative scores are consistent with a core essential function across cell lines.

**
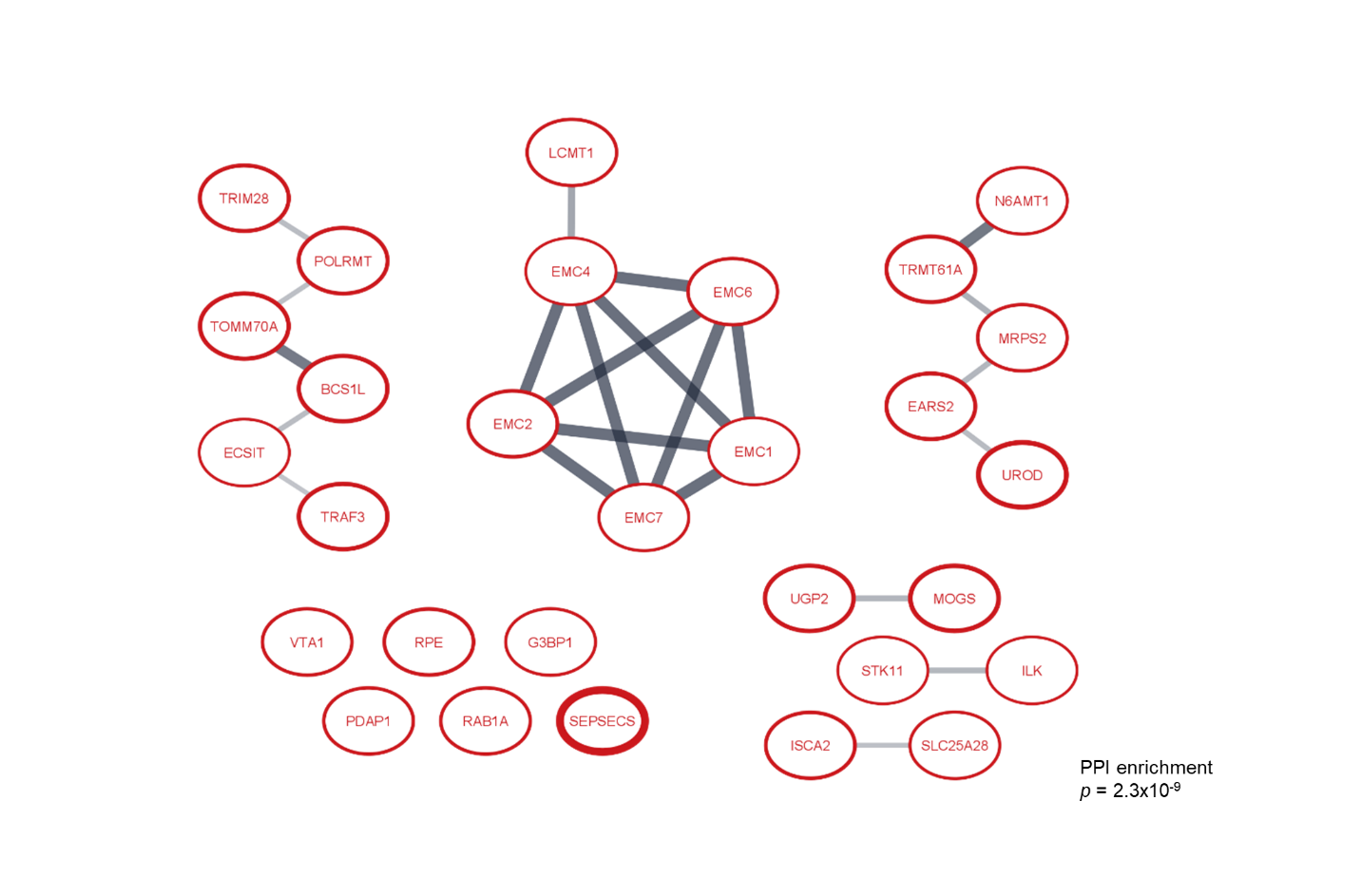
Figure S4. Established networks among genes whose disruption reduced the fitness of Calu-3 cells during SARS-CoV-2 infection.** Borders of individual nodes are weighted by the -log(RRA score) in the screen, and lines connecting nodes are weighted by the strength of the protein-protein interaction within the STRING database. The significance of the number of protein-protein interactions relative to a randomly selected gene set was calculated by STRING.

**
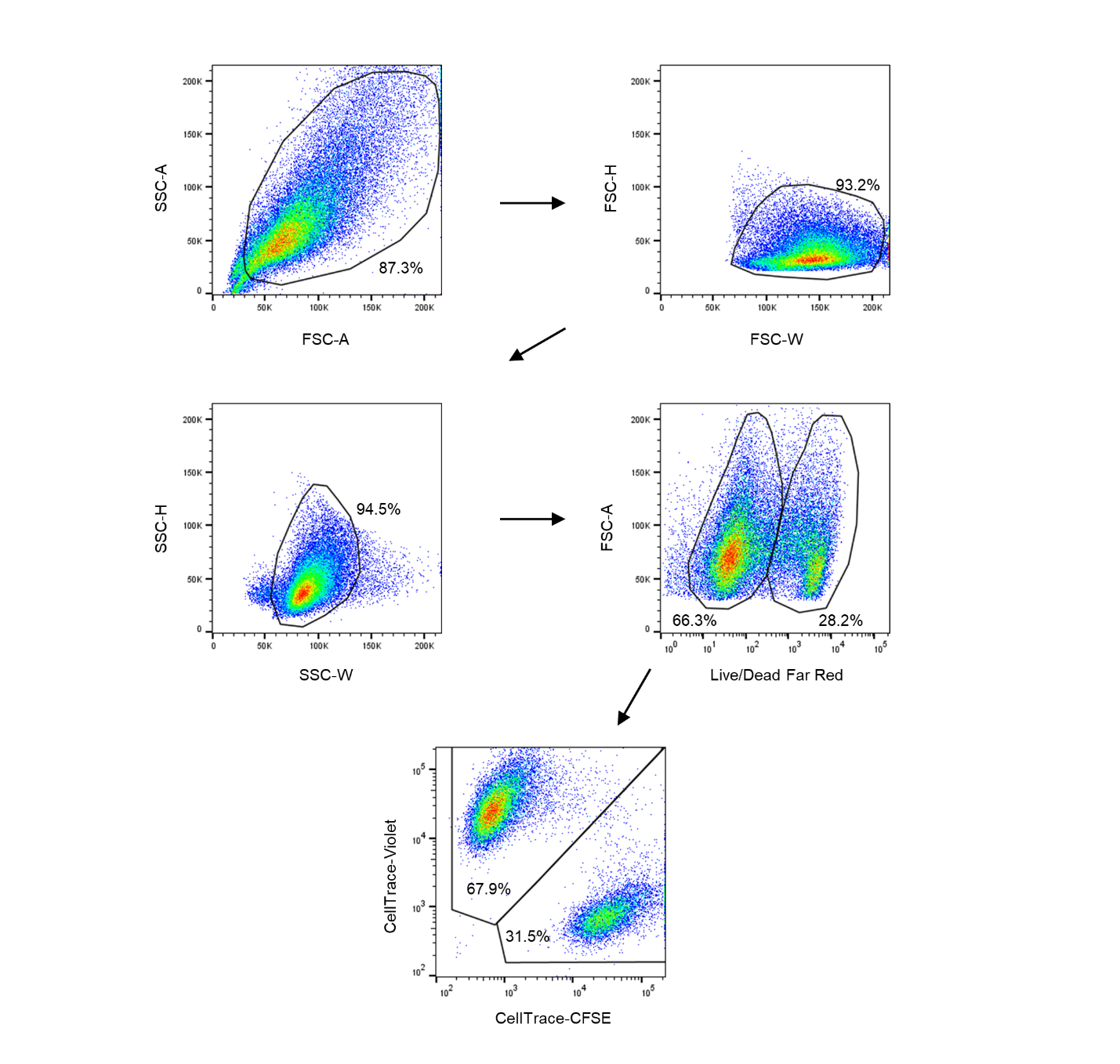
Figure S5. Representative flow cytometry plots**. Gating strategy for quantifying relative proportions of cells labeled with CellTrace-CFSE and CellTrace-Violet, as indicated in Figures 2, 4, 5, 6, and S6.

**
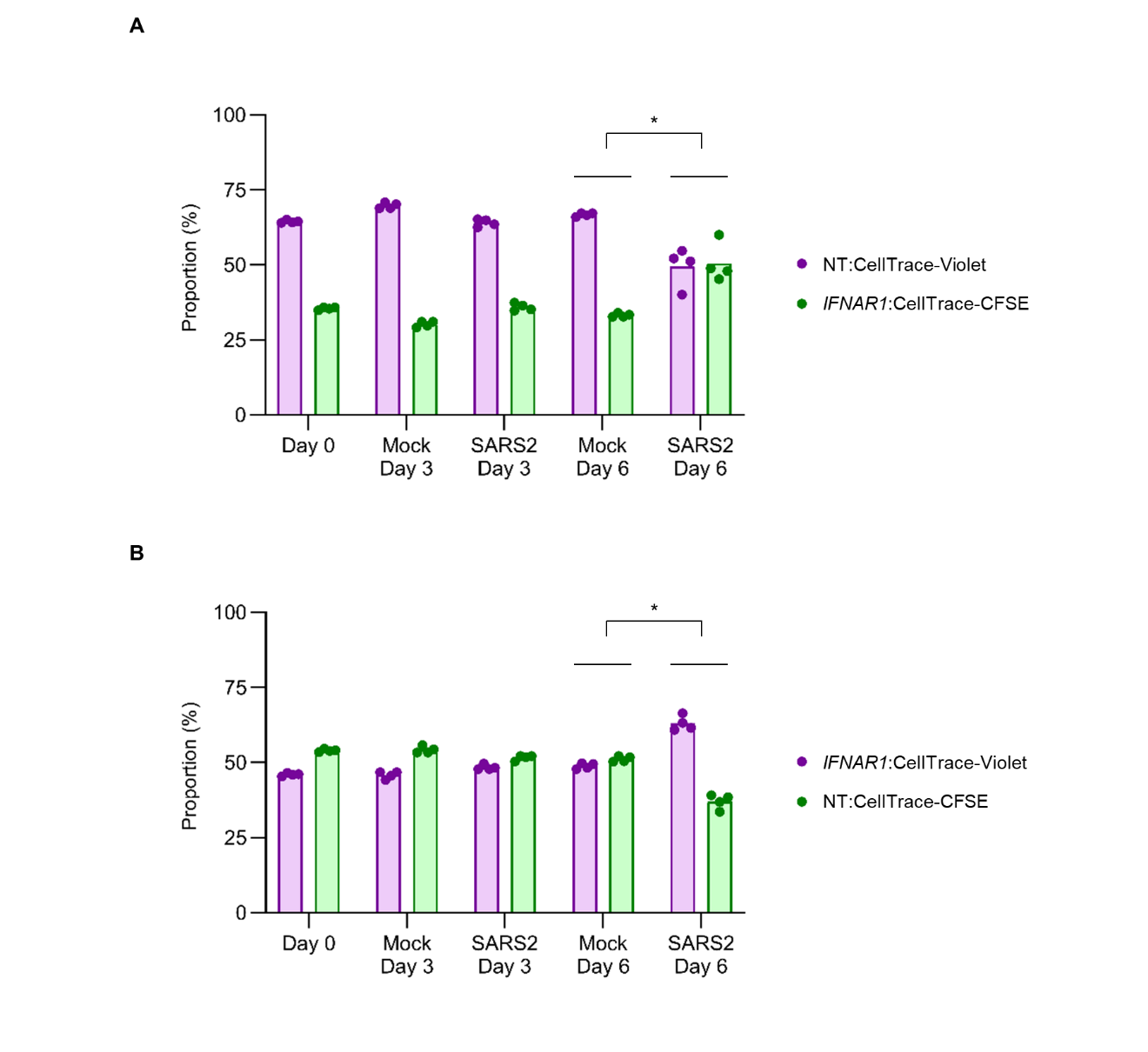
Figure S6. The competitive advantage of *IFNAR1*-disrupted cells in co-culture with control nontargeting gRNA-treated cells during SARS-CoV-2 infection is not mediated by the fluorescent tracker.** Calu-3 cells treated with a nontargeting gRNA were labeled with either CellTrace-Violet (A) or CellTrace-CFSE (B) and seeded in co-culture with cells treated with a *IFNAR1*-targeting gRNA and labeled with the other fluorescent tracker, CellTrace-CFSE (A) or CellTrace-Violet (B). The relative abundance of each cell population was analyzed by flow cytometry at the indicated time points under conditions of mock or SARS-CoV-2 infection. Data points represent independent infections and asterisks indicate p < 0.05 by Student’s t-test for the relative proportions of each population between SARS-CoV-2-infected and mock-infected cells.

**
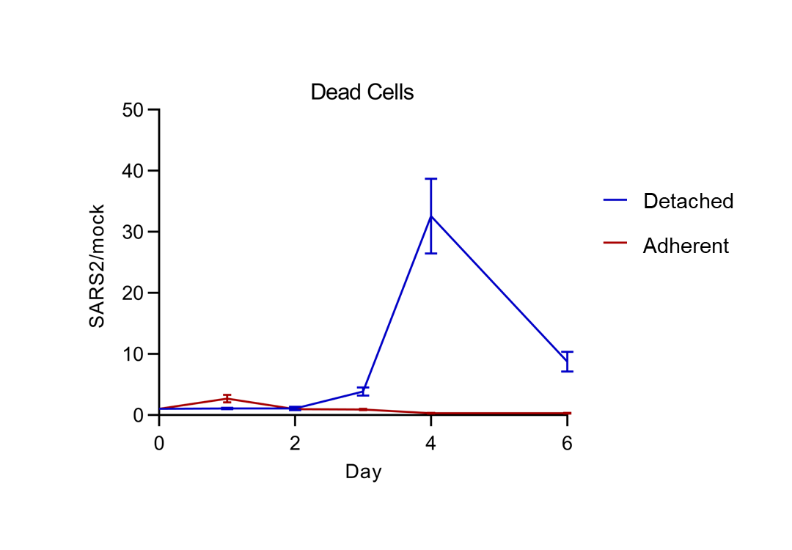
 Figure S7. Quantification of adherent and detached dead cells over the course of mock or SARS-CoV-2 infection.** The ratio of dead cells associated with either the cell monolayer or detached in the conditioned media is plotted for SARS-CoV-2 infection relative to mock infection conditions at the indicated time points. Error bars represent standard deviation of 4 independent infections.

**
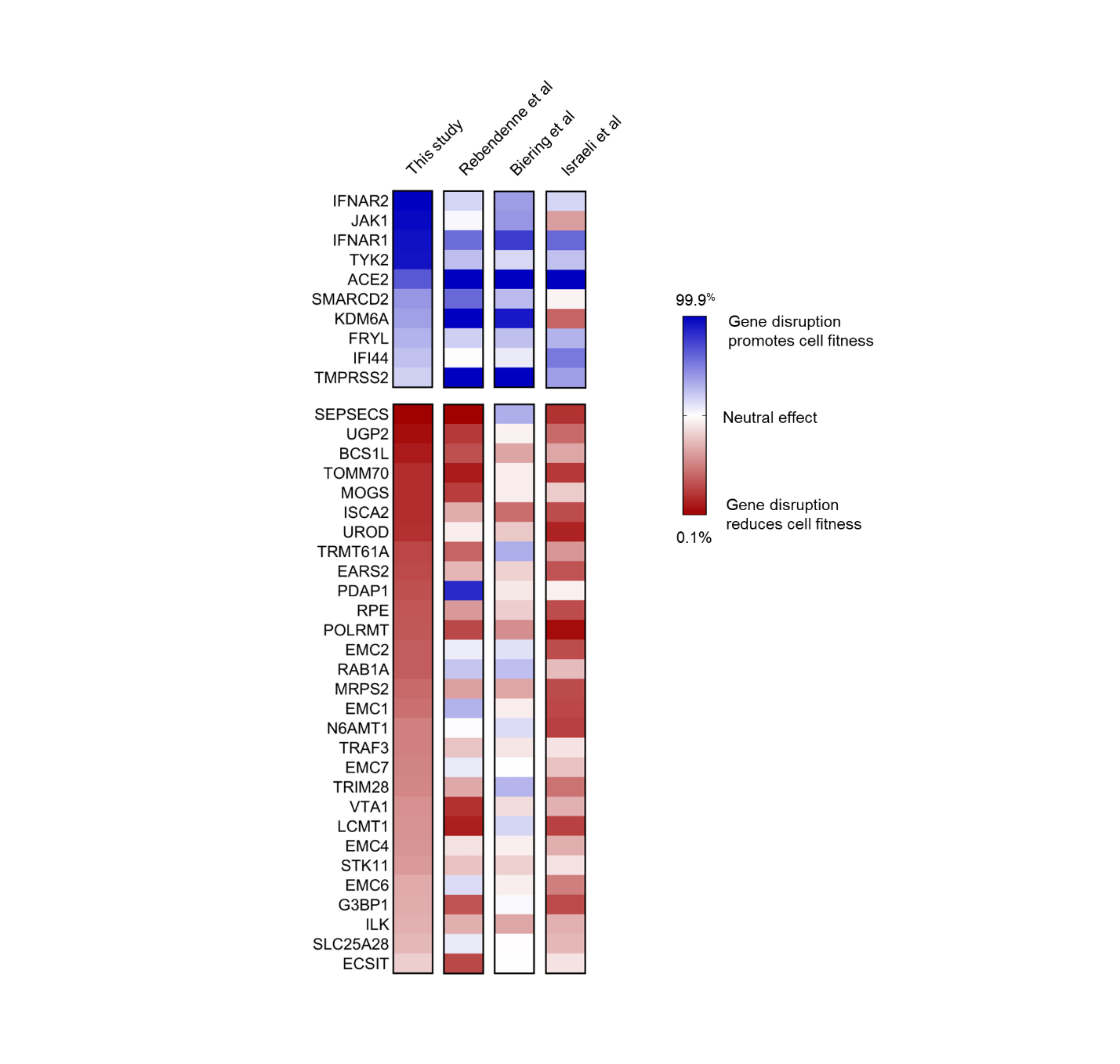
Figure S8. Comparative analysis of genes identified in this study with other published CRISPR screens of Calu-3 cell fitness during SARS-CoV-2 infection.** Heat map of aggregate gRNA enrichment (blue) or depletion (red) for the indicated genes in each CRISPR screen. Values from other published studies were normalized with the end of each color spectrum representing the 99.9 and 0.1 percentiles among all genes queried in the screen, placed in rank order by log2FC or Z-score.

**
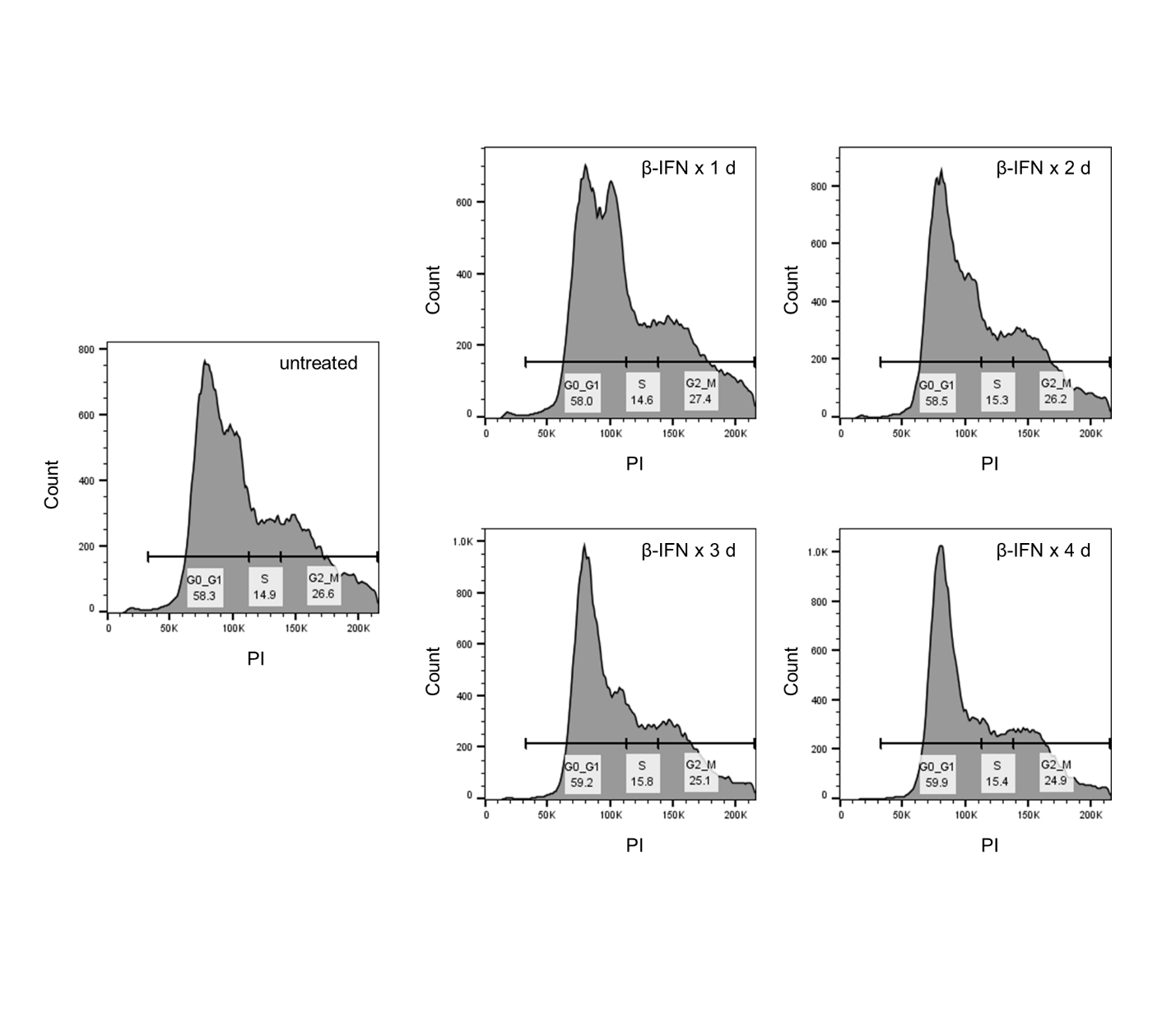
Figure S9. Cell cycle analysis of β-IFN-treated Calu-3 cells.** Flow cytometry histograms of propidium iodide staining of Calu-3 cells over the course of 4 days of treatment with 1000 U/mL exogenous β-IFN**.**

**Supplemental Table 1. Gene-level CRISPR screen results for modifiers of Calu-3 cell fitness during SARS-CoV-2 infection.** MAGeCK output for aggregate gene-level gRNA-level enrichment or depletion following SARS-CoV-2 infection relative to input cells. Negative log_2_-fold change and RRA scores indicate gRNA depletion in SARS-CoV-2-infected cells (gene disruption reduces cell fitness); positive values indicated gRNA enrichment in SARS-CoV-2-infected cells (gene increases cell fitness). Genes highlighted in red represent genes whose disruption had a significant impact on Calu-3 cell fitness in the absence of viral infection (Fig S3, Supplemental Tables 3-4).

**Supplemental Table 2. Individual gRNA-level CRISPR screen results for modifiers of Calu-3 cell fitness during SARS-CoV-2 infection.** MAGeCK output for individual gRNA-level enrichment or depletion following SARS-CoV-2 infection relative to input cells. Negative log_2_-fold change and RRA scores indicate gRNA depletion in SARS-CoV-2-infected cells (gene disruption reduces cell fitness); positive values indicated gRNA enrichment in SARS-CoV-2-infected cells (gene increases cell fitness). Genes highlighted in red represent genes whose disruption had a significant impact on Calu-3 cell fitness in the absence of viral infection (Fig S3, Supplemental Tables 3-4).

**Supplemental Table 3. Gene-level CRISPR screen results for modifiers of the fitness of uninfected Calu-3 cells.** MAGeCK output for aggregate gene-level gRNA-level enrichment or depletion in cells transduced with lentiviral pool for 2-3 weeks prior to onset of SARS-CoV-2 infection, relative to plasmid pool. Negative log_2_-fold change and RRA scores indicate gRNA depletion in SARS-CoV-2-infected cells (gene disruption reduces cell fitness); positive values indicated gRNA enrichment in SARS-CoV-2-infected cells (gene increases cell fitness).

**Supplemental Table 4. Individual gRNA-level CRISPR screen results for modifiers of the fitness of uninfected Calu-3 cells.** MAGeCK output for individual gRNA-level enrichment or depletion in cells transduced with lentiviral pool for 2-3 weeks prior to onset of SARS-CoV-2 infection, relative to plasmid pool. Negative log_2_-fold change and RRA scores indicate gRNA depletion in SARS-CoV-2-infected cells (gene disruption reduces cell fitness); positive values indicated gRNA enrichment in SARS-CoV-2-infected cells (gene increases cell fitness).

**Supplemental Table 5. Statistical analysis.** Source data and statistical analyses for the indicated figures.
